# SHISA3 Reprograms Tumor‐Associated Macrophages Toward an Antitumoral Phenotype and Enhances Cancer Immunotherapy

**DOI:** 10.1002/advs.202403019

**Published:** 2024-07-25

**Authors:** Shimeng Zhang, Bingbing Yu, Chunjie Sheng, Chen Yao, Yang Liu, Jing Wang, Qi Zeng, Yizhi Mao, Jinxin Bei, Bin Zhu, Shuai Chen

**Affiliations:** ^1^ State Key Laboratory of Oncology in South China Guangdong Provincial Clinical Research Center for Cancer Sun Yat‐sen University Cancer Center Guangzhou 510060 P. R. China; ^2^ Key Laboratory of Molecular Biophysics the Ministry of Education College of Life Science and Technology Huazhong University of Science and Technology Wuhan Hubei 430074 P. R. China; ^3^ Shenzhen Huazhong University of Science and Technology Research Institute Shenzhen 518063 P. R. China

**Keywords:** cancer immunotherapy, macrophage polarization, SHISA3, TAMs

## Abstract

The main challenge for immune checkpoint blockade (ICB) therapy lies in immunosuppressive tumor microenvironment (TME). Repolarizing M2‐like tumor‐associated macrophages (TAMs) into inflammatory M1 phenotype is a promising strategy for cancer immunotherapy. Here, this study shows that the tumor suppressive protein SHISA3 regulates the antitumor functions of TAMs. Local delivery of mRNA encoding Shisa3 enables cancer immunotherapy by reprogramming TAMs toward an antitumoral phenotype, thus enhancing the efficacy of programmed cell death 1 (PD‐1) antibody. Enforced expression of Shisa3 in TAMs increases their phagocytosis and antigen presentation abilities and promotes CD8^+^ T cell‐mediated antitumor immunity. The expression of SHISA3 is induced by damage/pathogen‐associated molecular patterns (DAMPs/PAMPs) in macrophages via nuclear factor‐κB (NF‐κB) transcription factors. Reciprocally, SHISA3 forms a complex with heat shock protein family A member 8 (HSPA8) to activate NF‐κB signaling thus maintaining M1 polarization of macrophages. Knockout Shisa3 largely abolishes the antitumor efficacy of combination immunotherapy with Toll‐like receptor 4 (TLR4) agonist monophosphoryl lipid A (MPLA) and PD‐1 antibody. It further found that higher expression of SHISA3 in antitumoral TAMs is associated with better overall survival in lung cancer patients. Taken together, the findings describe the role of SHISA3 in reprogramming TAMs that ameliorate cancer immunotherapy.

## Graphical abstract

This study identified SHISA3 as a valuable therapeutic gene for cancer immunotherapy. Higher expression of SHISA3 in TAMs is associated with better overall survival in lung cancer patients. Enforced expression of Shisa3 in TAMs enables cancer immunotherapy by NF‐κB‐mediated reprogramming of TAMs toward an antitumoral phenotype, thus enhances the efficacy of immune checkpoint blockade (ICB) therapy.

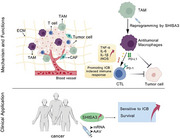

## 1 Introduction

Immunotherapies have been viewed as the most attractive therapeutic strategy for cancer.^[^ ^1^, ^2^ ^]^ Blocade antibodies targeting immune checkpoints cytotoxic T lymphocyte‐associated antigen 4 (CTLA‐4) or PD‐1 have shown definite long‐term antitumor activity in ≈30% of solid tumor patients, but their efficacy is limited for most patients.^[^ ^3^ ^]^ One possible reason is the immunosuppressive components of tumor microenvironment (TME).^[^ ^4^ ^]^ The TME comprises cancer associated fibroblasts (CAFs)^[^ ^5^ ^]^ and suppressive immune cells, including myeloid suppressor cells (MDSCs),^[^ ^6^ ^]^ tumor associated macrophages (TAMs),^[^ ^7^ ^]^ tumor associated neutrophils (TANs),^[^ ^8^ ^]^ mast cells^[^ ^9^ ^]^ and regulatory T cells (Tregs),^[^ ^10^ ^]^ which contribute to immunosuppression and drug resistance.

TAMs are one of the most abundant tumor infiltrating immune cells in various types of tumors. As a result of the phenotypic plasticity, macrophages can be polarized into M1 and M2 type.^[^ ^11^ ^]^ M2 macrophages, as the main type of TAMs in TME, play a critical role in angiogenesis, tumor cell metastasis, tumor stem cell formation, energy metabolism, and immunosuppression.^[^ ^7^, ^12^, ^13^ ^]^ On the contrary, M1 macrophages exert the function of tumor killing cells by recognizing and engulfing tumor cells, producing pro‐inflammatory cytokines such as tumor necrosis factor *α* (TNF*α*), interleukins‐1*β* (IL‐1*β*), IL‐6, IL‐12, and IL‐23 that promote the recruitment of adaptive immune cells, and upregulating inducible nitric oxide synthase (iNOS) to metabolize arginine into the nitric oxide (NO).^[^ ^14^, ^15^ ^]^ Reprograming M2‐like TAMs into M1 macrophages is a promising strategy in anti‐tumor therapy.^[^ ^16^ ^]^ Chimeric antigen receptor macrophages (CAR‐Ms) transduced with adenovirus vectors^[^ ^17^ ^]^ or generated with new CAR structure including toll/IL‐1R (TIR) signal transduction domain^[^ ^18^ ^]^ maintain the M1 phenotype and show significant anti‐tumor activity. Further elucidating the molecular mechanisms of macrophage polarization that promote the transformation of TAMs into M1 macrophages undoubtedly has the potential to enhance cancer immunotherapy.

SHISA3, a member of the SHISA family,^[^ ^19^ ^]^ is a transmembrane protein that has been reported to inhibit the Wnt/*β*‐catenin signaling pathway and functions as a tumor suppressor.^[^ ^20^, ^21^ ^]^ Hypermethylation of *SHISA3* promoter occurs in a variety of tumors, including breast cancer, (BC)^[^ ^22^ ^]^ colorectal cancer (CRC),^[^ ^23^ ^]^ nasopharyngeal cancer (NPC),^[^ ^24^ ^]^ and lung adenocarcinoma (LUAD).^[^ ^25^, ^26^ ^]^ However, its role in immune cells, including macrophages, is not yet revealed. In this study, we found that damage‐associated molecular patterns (DAMPs) and pathogen‐associated molecular patterns (PAMPs) induce SHISA3 expression through the NF‐κB pathway in macrophages. SHISA3 forms an immune complex with HSPA8 and reciprocally activates NF‐κB axis to promote macrophage M1 polarization. Local injection of AAV‐Shisa3 transducted macrophages or nanoparticle delivered Shisa3 mRNA significantly enhanced the efficacy of anti‐PD‐1 therapy through promoting the infiltration of CD8 cytotoxic T cells in TME. These results indicate a new function of SHISA3 in regulating macrophage polarization, and suggest SHISA3 as an attractive target for cancer immunotherapy.

## 2 Results

### 2.1 Shisa3 Promotes Antitumor Immunity

**Figure 1:**
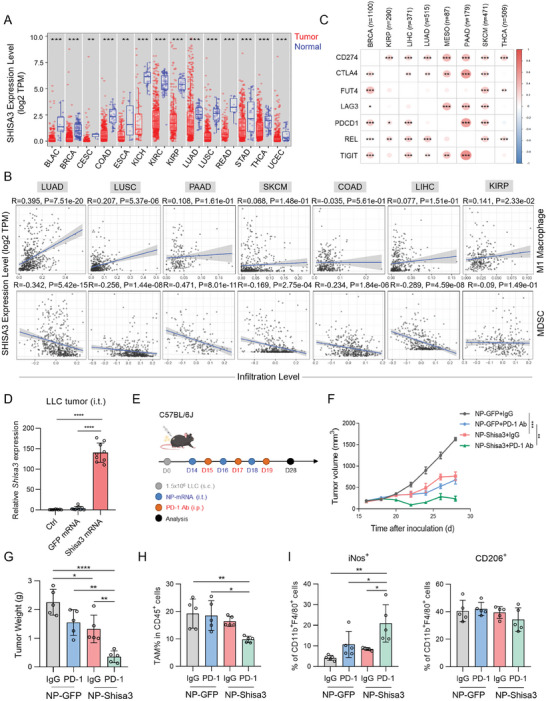
SHISA3 promotes antitumor immunity. A) TCGA database analysis of SHISA3 mRNA expression levels in primary tumors and normal tissues. B) Correlation SHISA3 expression and M1‐type macrophage infiltration (up) or MDSC infiltration (down) by TIMER 2.0 website (http://timer.cistrome.org/). C) The bubble plots show the positive correlation of SHISA3 expression with immune‐related markers in different types of cancer. D) qRT‐PCR of *Shisa3* expression of LLC tumors transduced with NP‐Shisa3 by intratumoral injections for 40 h. E) Schematic diagram showing s.c. injection of LLC cells and the treatment plan (Created with gdp.renlab.cn). F,G) LLC tumor growth (F) and weight (G) on day 28 after tumor inoculation were treated with intratumoral injection of NP‐GFP or NP‐Shisa3 in combination with i.p. injection of IgG or anti‐PD‐1. H,I) Flow cytometry assay of TAM percentages (H), percentage of iNos^+^ (I left) and CD206^+^ (I right) TAMs in LLC tumors treated with NP‐GFP or NP‐Shisa3 in combination with IgG or anti‐PD‐1. Data are presented as mean ± SD and were analyzed by one‐way ANOVA (D and G‐I) or two‐way ANOVA with a mixed‐effects model and adjusted by Holm–Šídák post‐hoc test (F). ^*^ *p* < 0.05, ^**^ *p* < 0.01, ^****^ *p* < 0.0001. Data are representative of at least two independent experiments. Abbreviation for TCGA cancer types: LUAD, Lung adenocarcinoma; LUSC, Lung squamous cell carcinoma; PAAD, Pancreatic adenocarcinoma; SKCM, Skin cutaneous melanoma; COAD, Colon adenocarcinoma; LIHC, Liver hepatocellular carcinoma; KIRP, Kidney renal papillary cell carcinoma; BRCA, Breast invasive carcinoma; THCA, Thyroid carcinoma.

We screened the expression of SHISA3 in tumors and adjacent normal tissues. The Cancer Genome Atlas (TCGA) database analysis showed that SHISA3 expression level was markedly downregulated in 14 solid tumor types (**Figure** **1**A), which is in agreement with previous reports.^[^ ^22^, ^23^, ^24^, ^25^, ^26^ ^]^ By analyzing gene expression correlation in LUAD, LUSC, PAAD, SKCM, COAD, LIHC, and KIRP using the TIMER 2.0 website, we found that higher expression of SHISA3 positively correlated with M1 type macrophage infiltration and negatively correlated with MDSC infiltration in most types of these cancers (Figure 1B). A positive correlation between SHISA3 and CD8^+^ T cells was also shown in most types of these cancers (Figure S1A, Supporting Information). For TAMs are the major components of the TME in LUAD^[^ ^27^ ^]^ and the positive correlation between SHISA3 level and M1 macrophage infiltration in LUAD is the most significant among these cancers (Figure 1B), we next explored the clinical significance of SHISA3 in LUAD patients. By using the ESTIMATE algorithm to develop a gene signature based on the estimation of stromal and immune cells in malignant tumor tissues, we found a positive correlation between the expression of SHISA3 and immune scores in several databases (Figure S1B, Supporting Information). In addition, we conducted a comprehensive analysis of the expression features of inhibitory receptors (IRs), expression of SHISA3 was positively correlated with the expression of immune checkpoint genes, such as CD274 (encoding PD‐L1), TIGIT, LAG3, CTLA4, and PDCD1 (encoding PD‐1) in a variety of types of cancer (Figure 1C).^[^ ^28^ ^]^ These results showed that tumor suppressor SHISA3 may play a critical role in antitumor immunity.

To examine whether overexpression of SHISA3 in the TME would be beneficial for the treatment of tumors in vivo, we used mRNA transfection^[^ ^29^ ^]^ by nanoparticles (NP‐GFP, NP‐Shisa3) to introduce Shisa3 by intratumoral administration (Figure S1C, Supporting Information). In vivo uptake assays showed that TAMs in Lewis lung carcinoma (LLC) tumors from C57BL/6J mice could take up NP‐GFP efficiently (Figure S1D,E, Supporting Information). qPCR analysis of cells isolated from LLC tumors that received intratumoral (i.t.) injection of NP‐Shisa3 showed that *Shisa3* mRNA was significantly higher than control mice (Figure 1D). For in vivo treatment, we combined mRNA therapy with PD‐1 antibody (Figure 1E). We found that NP‐Shisa3 alone could reduce tumor growth compared with NP‐GFP treatment, and combined with anti‐PD‐1 displayed the strongest antitumor efficacy (Figure 1F,G). The weight of the mice did not change significantly (Figure S1F, Supporting Information). We next analyzed the contributions of immune cells to antitumor activity. By flow cytometry (Figure S2A, Supporting Information), we found that NP‐SHISA3 injection combined with anti‐PD‐1 resulted in a twofold decrease in TAMs and a fivefold increase in CD11b^+^ F4/80^+^ iNos^+^ antitumoral macrophages in LLC‐bearing mice (Figure 1H,I). These results suggest that local delivery of Shisa3 mRNA by nanoparticles promotes antitumor immunity.

### 2.2 SHISA3 Enforced Expression in BMDMs Enhances the Efficacy of PD‐1 Blockade

To address the potential role of SHISA3 in TAMs during tumor development, we first try to overexpress Shisa3 in macrophages. We compared the infection efficiency of lentivirus, adeno‐associated virus serotype 1 (AAV1), and AAV2 with the same titer. By detecting the expression of GFP, we found that AAV1 has the highest transduction efficiency (≈90%) in bone‐marrow‐derived macrophages (BMDMs) (Figure S2B, Supporting Information; the term AAV used in this study specifically refers to AAV1), and after AAV‐Shisa3 transduction, the Shisa3 was overexpressed successfully in BMDMs (Figure S2C, Supporting Information). Cytoplasm and membrane localization of Shisa3 was also detected by immunofluorescence staining (Figure S2D, Supporting Information).

**Figure 2:**
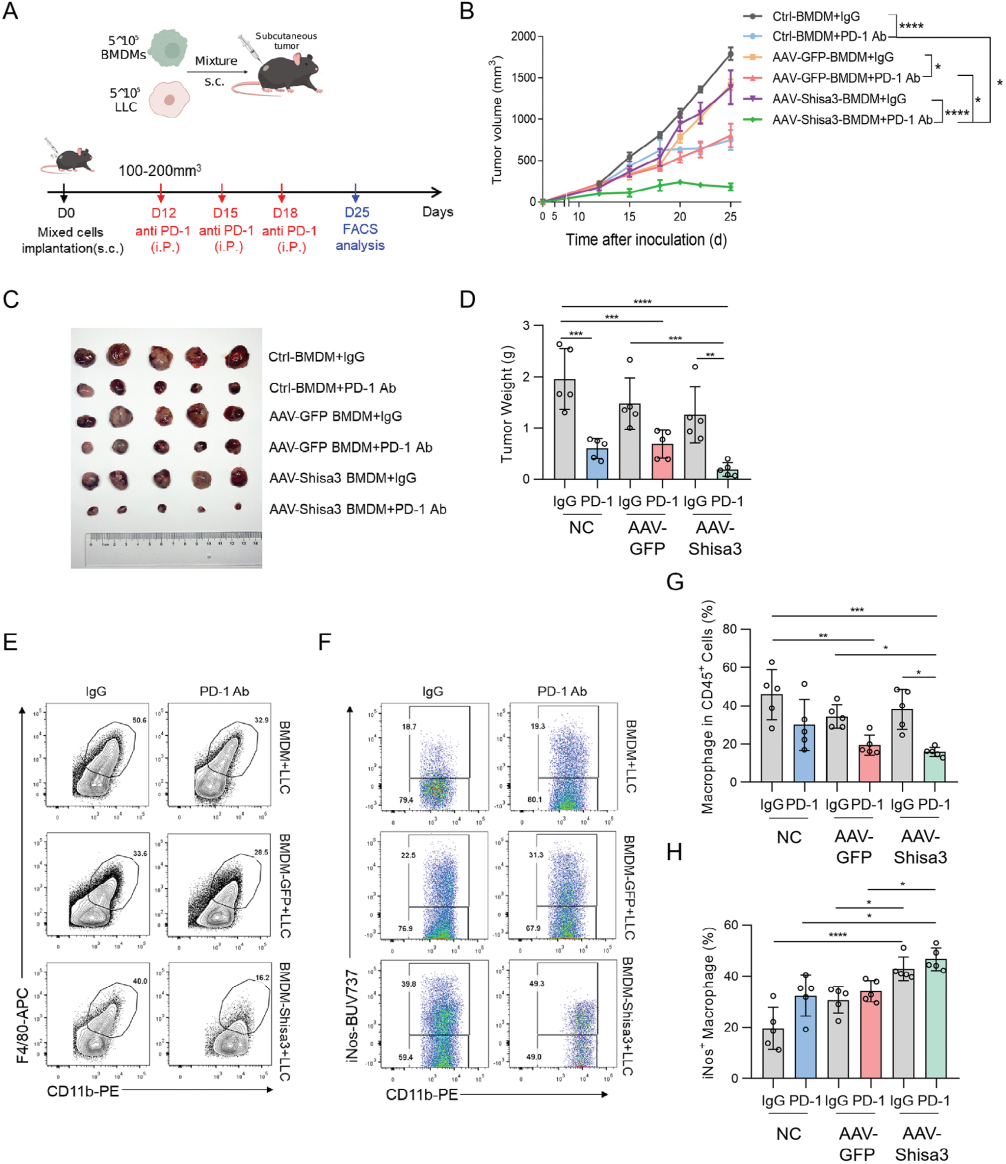
SHISA3 overexpression in BMDMs suppresses tumor growth and enhances the efficacy of PD‐1 blockade. A) Schematic diagram showing s.c. co‐injection of LLC cells with BMDMs (above) and the treatment plan (down) in LLC tumor‐bearing mice (Created with gdp.renlab.cn). B–D) LLC tumor growth (B), image (C) and weight (D) on day 25 after s.c. co‐injection of LLC mixed with control BMDMs, AAV‐Shisa3 or AAV‐GFP transduced BMDMs in C57BL/6 mice in combination with i.p. injection of IgG or anti‐PD‐1. E,F) Dot‐plots of TAM (E) and iNos staining (F) in TAMs in LLC tumors. G,H) Percentages of TAM (G) and iNos^+^ TAMs (H) in LLC tumors. Data are presented as mean ± SD and were analyzed by one‐way ANOVA (D, G, H) or two‐way ANOVA with a mixed‐effects model and adjusted by Holm–Šídák post‐hoc test (B). ^*^ *p* < 0.05, ^**^ *p* < 0.01, ^***^ *p* < 0.001, ^****^ *p* < 0.0001. Data are representative of at least two independent experiments.

Next, we designed subcutaneously co‐inoculation of murine BMDMs with LLC models to test the antitumor effect of Shisa3 (**Figure** **2**A). We observed that the combination therapy group (AAV‐Shisa3 transduced BMDMs plus anti‐PD‐1) significantly reduced tumor growth compared with the monotherapy groups (Figure 2B–D). In addition, flow cytometry indicated that the percentages of TAMs were decreased in the combination therapy group (AAV‐Shisa3 transduced BMDMs injection combined with PD‐1 antibody) compared with the monotherapy group (AAV‐Shisa3 transduced BMDMs injection or control BMDMs injection combined with PD‐1 antibody), and the percentages of CD11b^+^ F4/80^+^ iNos^+^ M1 type TAMs were markedly increased in the combination therapy group on day 25 after inoculation (Figure 2E–H). It also showed that co‐inoculation of LLC with AAV‐Shisa3 transduced BMDMs significantly increased anti‐tumor TAMs more than untransduced BMDMs (Figure 2H). These results suggested that enforced expression of SHISA3 in macrophages could reprogram TAMs to M1 phenotype and showed a synergistic antitumor effect with anti‐PD‐1 therapy.

### 2.3 SHISA3 Enhances CD8^+^ T Cell‐Mediated Antitumor Immunity

**Figure 3:**
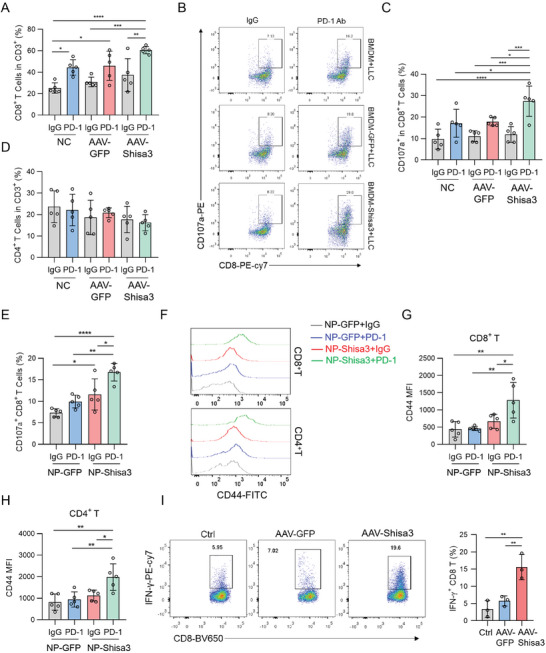
SHISA3 overexpression in BMDMs enhances CD8^+^ T cell‐mediated antitumor immunity. A) Flow cytometry assay of CD8^+^ T cell percentages in LLC tumors. B,C) Flow cytometry assay of CD107a staining (B) in CD8^+^ T cells and CD107a^+^ CD8^+^ T cells percentages (C) in LLC tumors. D) Flow cytometry assay of CD4^+^ T cell percentages in LLC tumors. E) Percentage of CD107a^+^ CD8^+^ T cells in LLC tumors treated with NP‐GFP or NP‐Shisa3 in combination with IgG or anti‐PD‐1. F–H) The overlaid histogram shows CD44 levels in CD4^+^ T or CD8^+^ T cells in LLC tumors treated with NP‐GFP or NP‐Shisa3 in combination with IgG or anti‐PD‐1. I) Representative dot‐plots (left) and the production of IFN‐*γ* (right) of CD8^+^ T cells co‐cultured with LLC and control BMDMs, AAV‐Shisa3 or AAV‐GFP transduced BMDMs treated with anti‐CD3 (5 µg mL^−1^) plus anti‐CD28 (5 µg mL^−1^) simultaneously for 72 h. Data are presented as mean ± SD and were analyzed by one‐way ANOVA. ^*^ *p* < 0.05, ^**^ *p* < 0.01, ^***^ *p* < 0.001, ^****^ *p* < 0.0001. Data are representative of at least three independent experiments.

Since a major activity of antitumoral TAMs is to promote antitumor immune responses and inhibit tumor progression, we then tested the overexpression of SHISA3 in BMDM on CD8^+^ T cell‐mediated antitumor immunity. Co‐inoculation of LLC with AAV‐Shisa3 transduced BMDMs then combined with PD‐1 antibody injection showed a striking increase in the percentages of infiltrating CD8^+^ T cells (**Figure** **3**A) and CD8^+^ cytotoxic T cells (Figure 3B,C) compared to other groups. There was no difference between the AAV‐GFP transduced BMDMs group with untransduced BMDMs therapy group (Figure 3A–C). The tumor‐infiltrating CD4^+^ T cells were comparable between all treatment groups (Figure 3D). In addition, when delivering *Shisa3* mRNA locally to tumors through nanoparticles, it also showed increased CD8 cytotoxic T cells in the combination therapy group (Figure 3E), and the T cell activation marker CD44 upregulated in the combination therapy group (Figure 3F–H). We then tested the effects of SHISA3 expression in macrophages on T‐cell activation in vitro. In BMDM and T cell co‐culture assays, we found that the production of IFN‐*γ* in CD8^+^ T cells was significantly increased in the AAV‐Shisa3 transduced BMDMs group, suggesting that the inhibitory effect of TAMs on T cells was alleviated (Figure 3I). Taken together, these results suggested that enforced expression of SHISA3 in macrophages or TME enhances CD8^+^ T cell‐mediated antitumor response.

### 2.4 SHISA3 Expression is Induced by NF‐κB Signaling

**Figure 4:**
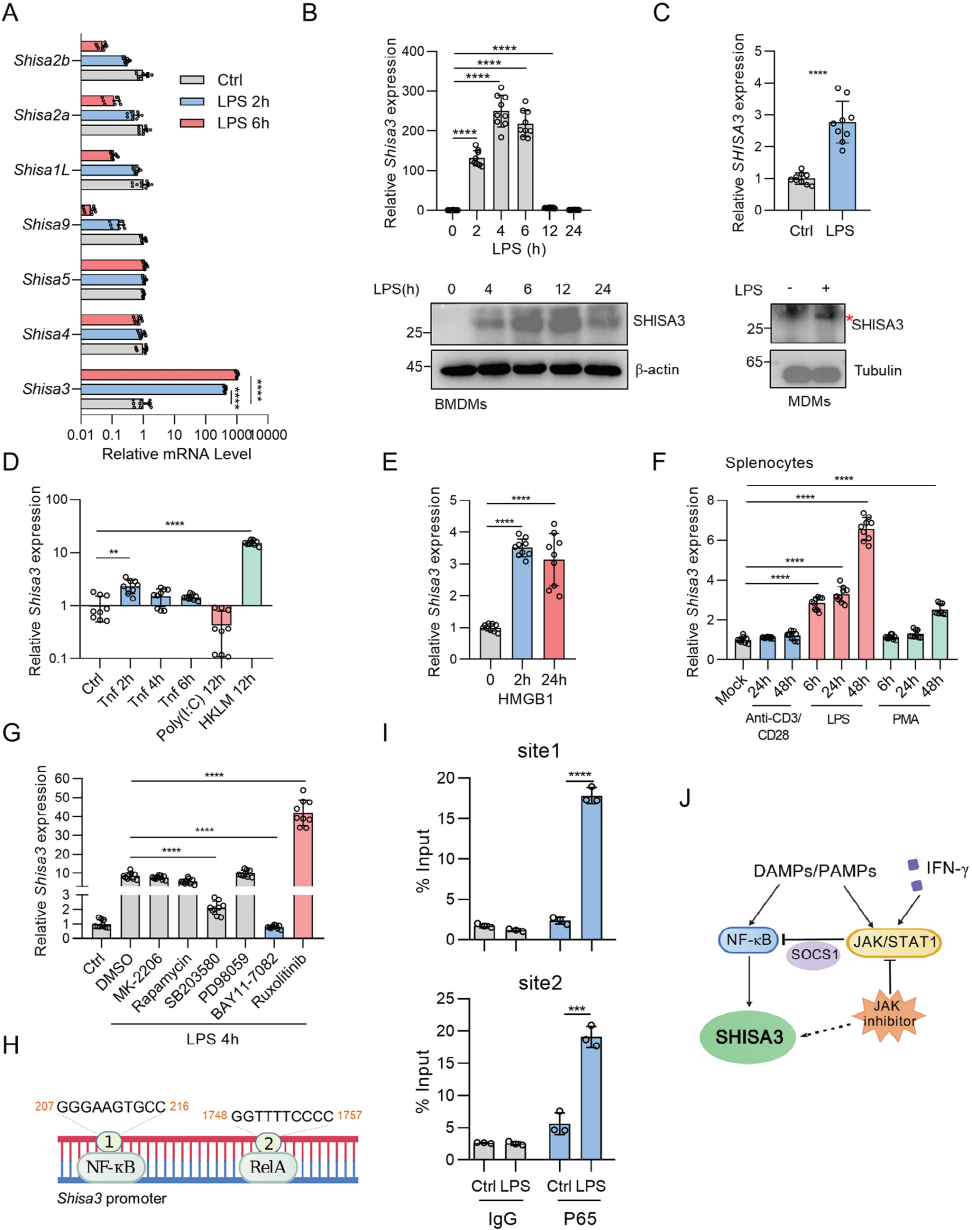
SHISA3 is induced by the PAMP/DAMP‐NF‐κB signaling pathway. A) qRT‐PCR of *Shisa* family molecules mRNA expression in BMDMs after LPS treatment at the indicated time point. B) qRT‐PCR of *Shisa3* (up) expression or Western blot of Shisa3 expression (down) in BMDMs after LPS stimulation. C) qRT‐PCR of *SHISA3* (up) expression or Western blot of SHISA3 expression (down) in MDMs after LPS stimulation. D) qRT‐PCR of *Shisa3* expression in BMDMs after stimulated with Tnf, poly (I: C), or HKLM. E) qRT‐PCR of *Shisa3* expression in BMDMs after stimulated with rmHMGB‐1 for 2, 24 h. F) qRT‐PCR of *Shisa3* expression in mouse splenocytes stimulated with anti‐CD3/CD28 antibody or LPS or PMA at the indicated time point. G) qRT‐PCR of *Shisa3* expression in BMDMs pretreatment with Akt inhibitor (MK‐2206), mTOR inhibitor (Rapamycin), p38 MAPK inhibitor (SB 203580), MEK inhibitor (PD98059), NF‐kB inhibitor (BAY 11‐7082), JAK1/2 inhibitor (Ruxolitinib), followed by LPS stimulation. H) PROMO Database predicts NF‐κB binding sites on *Shisa3* promoter. I) ChIP‐qPCR assay evaluates the enrichment of p65 on *Shisa3* promoter in BMDMs upon LPS stimulation. J) A Proposed model for how DAMPs/PAMPs regulate Shisa3 expression (Created with gdp.renlab.cn). Data are presented as mean ± SD and were analyzed by one‐way ANOVA (A, B and D‐G) or unpaired two‐tailed t‐test (C, I). ^**^ *p* < 0.01, ^***^ *p* < 0.001, ^****^ *p* < 0.0001. Data are representative of at least three independent experiments.

Hypermethylation of SHISA3 promoter leads to low expression of SHISA3 in many tumors.^[^ ^22^, ^23^, ^24^, ^25^, ^26^ ^]^ To investigate the expression pattern of SHISA3, we first explored the Shisa3 expression in normal tissues and cells through databases, the results showed that Shisa3 is highly expressed in BMDMs treated with LPS for 6 h, and still very low in other cells (Figure S3A, Supporting Information). Then transcriptomic analysis of LPS stimulated BMDMs showed that *Shisa3* was significantly upregulated in BMDMs stimulated with LPS for 4 h (Figure S3B, Supporting Information). We validated the expression of all Shisa family members and found that *Shisa3* is the only one that can be induced by LPS (**Figure** **4**A). After LPS stimulation, the Shisa3 level reached a peak at 4 h at the mRNA level or 12 h at the protein level (Figure 4B; Figure S3C, Supporting Information). The expression of SHISA3 was also significantly upregulated in human monocyte‐derived macrophages (MDMs) (Figure 4C) and THP‐1 monocytes, and J774A.1 mouse macrophages (Figure S3D, Supporting Information). Moreover, we did not detect significant differences in the expression of *Shisa3* during the polarization process of M2 macrophages induced by Il‐4 (Figure S3E,F, Supporting Information). In addition, the *Shisa3* level in macrophages was significantly increased after being treated with Tnf, poly (I: C), heat‐killed Listeria monocytogenes (HKLM), and HMGB1 (Figure 4D,E). In splenocytes, the expression of *Shisa3* is also significantly induced by LPS (Figure 4F). In the mouse model of LPS induced sepsis, the expression of *Shisa3* in bone marrow cells was greatly upregulated (Figure S3G, Supporting Information). Together, these data suggested that the expression of shisa3 was induced by DAMP/PAMPs.

To explore the molecular mechanism of LPS induced SHISA3 expression, we utilized multiple signaling pathway inhibitors to treat BMDMs and detected which pathways regulate Shisa3 expression during M1 macrophage polarization. In particular, we observed that NF‐κB inhibitor (BAY 11‐7082) significantly restrained the effect of LPS in upregulating *Shisa3* expression in macrophages, while *Shisa3* was further induced by LPS after using a JAK inhibitor (Ruxolitinib) (Figure 4G; Figure S4A,B, Supporting Information). For NF‐κB pathway, PROMO database predicted two NF‐κB binding sites in the *Shisa3* promoter (Figure 4H). Chromatin immunoprecipitation‐quantitative PCR (ChIP‐qPCR) assay confirmed that LPS treatment increased the enrichment of NF‐κB transcriptional factor p65 in the *Shisa3* promoter (Figure 4I). We also detected the *Shisa3* expression in BMDMs after LPS ± IFN‐*γ* (or OVA Peptide stimulated OT‐1 mouse CD8^+^ T cell supernatant, TSN) treatment, the results showed that LPS induced *Shisa3* expression was inhibited by IFN‐*γ* or TSN (Figure S4C,D, Supporting Information).

Next, we try to investigate the reason why the pre‐inhibition of JAK pathway potentiates while IFN‐*γ* or TSN treatment inhibits LPS induced *Shisa3* expression. We detected the increased expression of suppressor of cytokine signaling 1 (*Socs1*) and *Socs3*, two downstream genes and negative regulators of JAK/STAT,^[^ ^30^ ^]^ in response to LPS, Tnf and IFN‐*γ* (Figure S4E–H, Supporting Information). It has been reported that SOCS1 is also a negative regulator of NF‐κB axis,^[^ ^31^, ^32^ ^]^ the results suggested that SOCS1 diminishes SHSIA3 expression in response to IFN‐*γ* and TSN. To verify this hypothesis, we knocked down Socs1 in the BMDMs with two specific small interfering RNA (siRNAs, Figure S4I, Supporting Information). We found that Socs1 knockdown further increased the expression of *Shisa3* in BMDMs in response to LPS (Figure S4J, Supporting Information). Collectively, our findings indicated that DAMPs/PAMPs induced SHISA3 expression in macrophages through NF‐κB signaling, which was attenuated by IFN‐*γ*/JAK/STAT through SOCS1 (Figure 4J).

### 2.5 SHISA3 Promotes Macrophage Polarize Toward an M1 Phenotype

**Figure 5:**
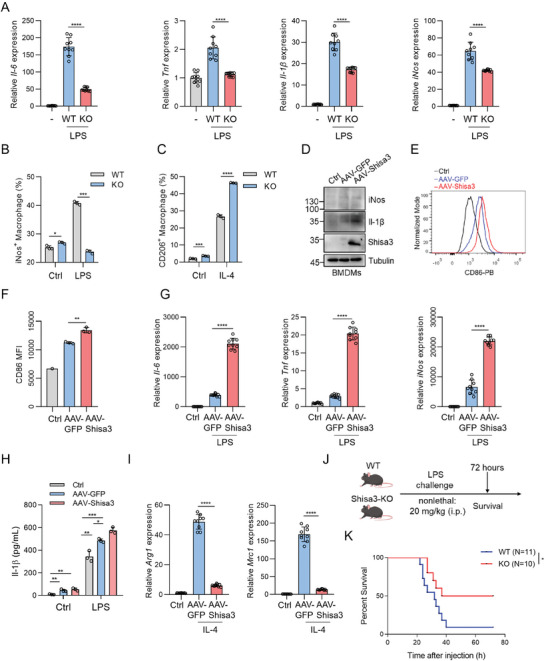
SHISA3 promotes macrophage polarization toward the M1 type. A) qRT‐PCR of M1 marker expression in WT and *Shisa3*‐KO BMDMs after LPS stimulation for 4 h. B) Flow cytometry assay of iNos expression in WT and *Shisa3*‐KO BMDMs after LPS stimulation for 24 h. C) Flow cytometry assay of the percentages of CD206^+^ macrophages in WT and *Shisa3*‐KO BMDMs after Il‐4 stimulation for 24 h. D) Western blot analysis of iNos, Il‐1*β*, and Shisa3 expression in BMDMs administered with or without AAV‐GFP or AAV‐Shisa3. E,F) Flow cytometry assay of CD86 level in Shisa3 overexpressing BMDMs or control BMDMs. G) qRT‐PCR of M1 marker expression in uninfected, AAV‐GFP or AAV‐Shisa3 infected BMDMs after LPS stimulation. H) Protein levels of Il‐1*β* in the supernatant of uninfected BMDMs or BMDMs infected with AAV‐Shisa3 or AAV‐GFP (Ctrl), followed by LPS stimulation for 24 h. I) qRT‐PCR of M2 marker expression in uninfected, AAV‐GFP or AAV‐Shisa3 infected BMDMs after Il‐4 stimulation. J) Schematic diagram showing WT and *Shisa3*‐KO mice were i.p. injected with LPS (Created with gdp.renlab.cn). K) Survival rates of WT and *Shisa3*‐KO mice challenged with LPS. Data are presented as mean ± SD and were analyzed by unpaired two‐tailed t‐test (A‐C and G, I) or one‐way ANOVA (F, H) or log‐rank (Mantel‐Cox) test of OS (K). ^*^ *p* < 0.05, ^**^ *p* < 0.01, ^***^ *p* < 0.001, ^****^ *p* < 0.0001. Data are representative of at least three independent experiments.

To investigate whether SHISA3 regulates macrophage polarization, we generated Shisa3 knockout mice (hereafter, *Shisa3*‐KO; Figure S5A, Supporting Information). The frequency or composition of macrophages, neutrophils, T cells, and B cells at a steady state was similar in age‐ and sex‐matched *Shisa3*‐KO and wild‐type (WT) mice (Figure S5B–D, Supporting Information). We observed reduced levels of proinflammatory cytokines (*Il‐6*, *Tfn*, *Il‐1β*) and M1 marker *iNos* in *Shisa3*‐KO BMDMs versus WT BMDMs induced by LPS (**Figure** **5**A,B; Figure S6A, Supporting Information). Next, we polarized BMDMs to M2 phenotype by Il‐4, and observed that the expression of M2 markers CD206 is significantly increased in the *Shisa3*‐KO BMDMs than in WT BMDMs (Figure 5C; Figure S6B, Supporting Information). Moreover, interfered Shisa3 expression in BMDMs using siRNAs (si‐Shisa3‐1/‐2) diminished LPS induced expression of *Il‐6*, *Tnf*, *Il‐1β* and *iNos* (Figure S6C,D, Supporting Information). These data demonstrated that SHISA3 might promote M1 polarization.

Consistent with previous results, we observed M1 markers (*iNos*, CD86, *Il‐6*, *Il‐1β*) were upregulated in the AAV‐Shisa3 transduced BMDMs compared with the control BMDMs under the M0 state (Figure 5D–F; Figure S6E, Supporting Information). When BMDMs were polarized to M1‐phenotype by LPS, AAV‐Shisa3 significantly induced higher expression of proinflammatory cytokines (*Il‐6, Tnf, Il‐1β*) and M1 marker *iNos* (Figure 5G,H; Figure S6F, Supporting Information). In vitro mRNA transfection results showed that the mean flourescence intensity of CD86 increased in NP‐Shisa3 transfected Raw264.7 (Figure S6G–I, Supporting Information). Conversely, the M2 markers *Arg1* and *Mrc1* were decreased in AAV‐Shisa3 transduced BMDMs induced by Il‐4 (Figure 5I). To further confirm the role of Shisa3 induced macrophage M1 polarization in acute inflammation, we established LPS‐sepsis mouse model with WT or *Shisa3*‐KO mice. The mortality of *Shisa3*‐KO mice was lower than WT mice (Figure 5J,K). These results indicated that SHISA3 programs macrophages into pro‐inflammatory M1 phenotype.

### 2.6 SHISA3 Promotes M1 Macrophage Polarization by Activating the NF‐κB Pathway

**Figure 6:**
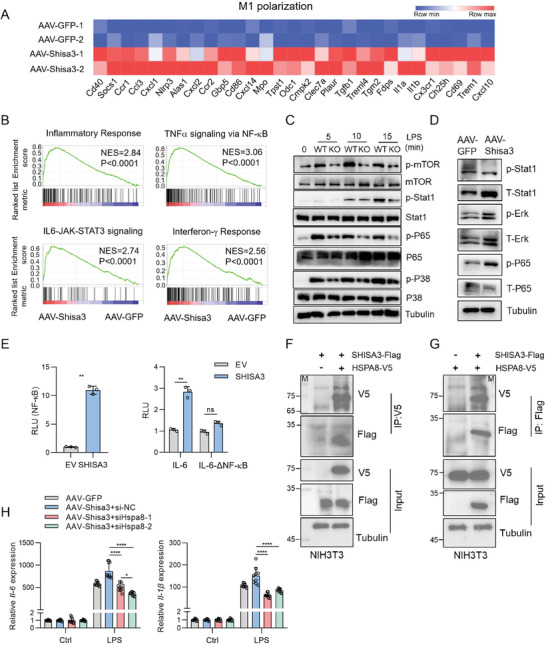
SHISA3 promotes macrophage M1 polarization through NF‐κB pathway. A) Heatmap showing mRNA abundance of M1 macrophage‐related genes in AAV‐Shisa3 transduced BMDMs versus control BMDMs. B) GSEA analysis of altered pathways between AAV‐Shisa3 and AAV‐GFP transduced BMDMs. C) Western blot analysis of NF‐κB, MAPKs (p38), mTOR, and JAK‐STAT1 activation in WT and *Shisa3*‐KO BMDMs after LPS stimulation. D) Western blot analysis of indicated pathways in AAV‐Shisa3 and AAV‐GFP transduced BMDMs. E) Dual luciferase reporter assays with NF‐κB reporter (left), IL‐6 reporter or IL‐6‐ΔNF‐κB reporter (right). F,G) Western blot analysis of interactions between Shisa3 and Hspa8 determined by coimmunoprecipitation with anti‐V5 (F) or anti‐Flag (G) antibody in NIH3T3 cells. H) qRT‐PCR of M1 marker expression in AAV‐Shisa3 or AAV‐GFP transduced BMDMs transfected with Hspa8 siRNA‐1/‐2 or control siRNA (si‐NC) after LPS stimulation for 4 h. Data are presented as mean ± SD and were analyzed by unpaired two‐tailed t‐test (E) or one‐way ANOVA (H). ^*^ *p* < 0.05, ^**^ *p* < 0.01, ^****^ *p* < 0.0001. Data are representative of at least three independent experiments.

We next identified SHISA3‐regulated signaling pathways by RNA sequencing (RNA‐seq) analysis of AAV‐Shisa3 transduced BMDMs and AAV‐GFP transduced BMDMs, and found that most M1 macrophage‐related genes were upregulated in the AAV‐Shisa3 transduced BMDMs (**Figure** **6**A). We further performed the Heatmap GO analysis on the Metascape website (http://metascape.org) to find out the key regulators. Interestingly, the results showed that overexpression of Shisa3 promotes biological processes such as macrophage immune response, cytokine production, cell migration, and activation, and the mainly affected genes were regulated by NF‐κB (Figure S7A,B, Supporting Information). Gene Set Enrichment Analysis (GSEA) further indicated a strong activation of the NF‐κB signaling pathway and inflammatory response in AAV‐Shisa3 transduced BMDMs (Figure 6B). Interestingly, GSEA showed that overexpression of Shisa3 also resulted in enrichment in the IL6‐JAK‐STAT3 signaling pathway and interferon‐*γ* response (Figure 6B), which was consistent with the aforementioned data. Western blots showed that knockout Shisa3 reduced the phosphorylation of NF‐κB, p38, mTOR, and STAT1, whereas NF‐κB was the most significantly activated pathway in AAV‐Shisa3 transduced BMDMs (Figure 6C,D; Figure S7C, Supporting Information). Luciferase reporter assays confirmed the activation of NF‐κB and IL‐6 reporter by SHISA3 (Figure 6E).

To further explore the molecular mechanism of NF‐κB regulation by Shisa3 in macrophages, we overexpressed Flag‐tagged Shisa3 in BMDMs and performed coimmunoprecipitation (Co‐IP) assay followed by mass spectrometry (MS) to identify the binding partners (Figure S7D–F, Supporting Information). We further validated the physiologic interaction between human SHISA3 and candidate interacting proteins in HEK‐293T cells (Figure S7G, Supporting Information). Hspa8, a protein reported to be associated with NF‐κB activation,^[^ ^33^ ^]^ was identified forming a complex with Shisa3 (Figure S7E–G, Supporting Information). Co‐IP analyses showed that Shisa3 interacted with Hspa8 in NIH‐3T3 cells (Figure 6F,G). Next, we sought to determine the function of the interaction between SHISA3 and HSPA8. The data showed knockdown of Hspa8 expression does not affect Shisa3 mRNA abundance in response to LPS treatment (Figure S7H–J, Supporting Information). After the knockdown of Hspa8, we found that the cytokine production induced by Shisa3 was reduced (Figure 6H), suggesting that Hspa8 mediates Shisa3‐induced NF‐κB activation in M1 polarization.

### 2.7 SHISA3 is a Therapeutic Target for Cancer Immunotherapy

**Figure 7:**
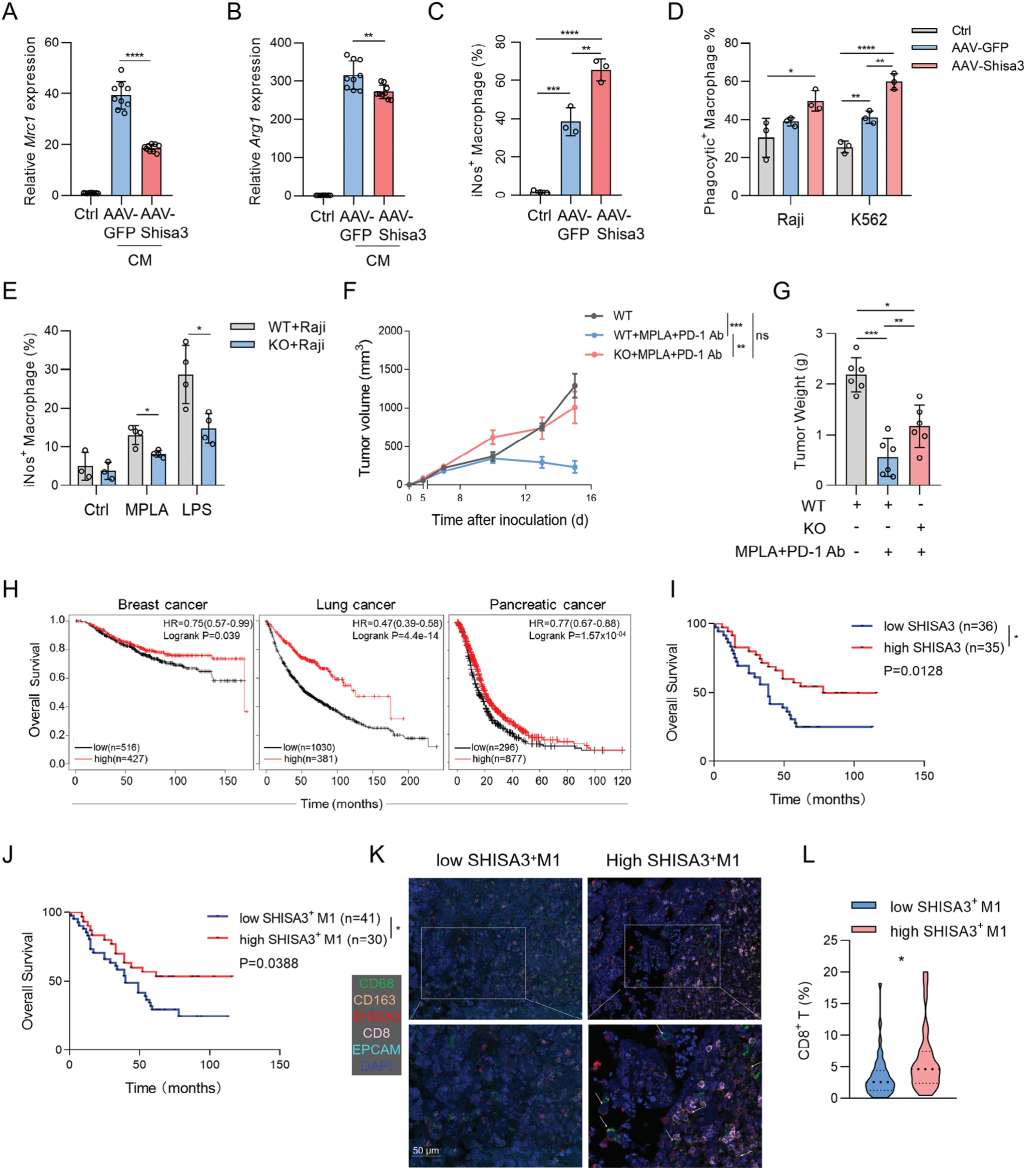
SHISA3 promotes TAM repolarization and displays a positive correlation with the Overall Survival of LUAD patients. A,B) qRT‐PCR of M2 markers *Mrc1* (A) and *Arg1* (B) in control BMDMs, AAV‐Shisa3 or AAV‐GFP transduced BMDMs after treatment with 4T1 cell‐CM. C) Scatterplots represent percentages of iNos staining in gated CD11b^+^ macrophages with or without AAV infection after treatment with LLC cell‐CM. D) Scatterplots represent percentages of phagocytosis of control BMDMs, AAV‐Shisa3 or AAV‐GFP transduced BMDMs for CellTrace Far Red labeled tumor cells (Raji or K562). E) Scatterplots represent percentages of iNos expression in WT BMDMs and Shisa3‐KO BMDMs co‐cultured with CellTrace™ Far Red labeled Raji cells. F,G) LLC tumor growth (F) and weight (G) on day 15 after tumor inoculation were treated with intratumoral injection of MPLA in combination with i.p. injection of anti‐PD‐1. H) Overall survival comparing high to low levels of SHISA3 in breast cancer, lung cancer, and pancreatic cancer patients by TIMER 2.0 website (http://timer.cistrome.org/). I,J) Overall survival comparing high to low levels of SHISA3 (I) or SHISA3 level in M1‐type TAMs (J) in 71 patients with NSCLC. K) mIHC of lung cancer patients. FFPE sections labeled with DAPI (blue), CD68 (green), CD163 (orange), CD8 (pink), EPCAM (cyan), and SHISA3 (red) were scanned using the Vectra imaging system. L) The frequencies of CD8^+^ T cells comparing high to low levels of SHISA3 in M1‐type TAMs in 71 patients with NSCLC. Data are presented as mean ± SD and were analyzed by one‐way ANOVA (C‐E, G), two‐way ANOVA with a mixed‐effects model and adjusted by Holm–Šídák post‐hoc test (F), log‐rank (Mantel‐Cox) test of OS (I, J) or unpaired two‐tailed t‐test (A, B, L). ^*^ *p* < 0.05, ^**^ *p* < 0.01, ^****^ *p* < 0.0001. Data are representative of at least two independent experiments.

To better mimic macrophages in the tumor microenvironment in vitro, we prepared conditioned medium (CM) derived from 4T1 cells or LLC, and then cultured BMDMs with CM to induce macrophage into TAMs. Our results indicated that compared to the blank control, the addition of CM significantly upregulated the expression of M2 macrophage markers *Arg1* and *Mrc1* mRNA in BMDMs, which was significantly attenuated with the enforced expression of Shisa3 (**Figure** **7**A,B). Moreover, the *Shisa3* level was decreased after treatment with CM (Figure S8A, Supporting Information). qPCR and flow cytometry (FACS) analysis showed that Shisa3 overexpressed BMDMs increased Il‐1*β* and iNos mRNA and protein expression in TAMs, suggesting that SHISA3 reprograms TAMs to the M1 subtype (Figure 7C; Figure S8B,C, Supporting Information).

Tumor cells overexpress “don't eat me” signals to escape phagocytosis by macrophages.^[^ ^34^ ^]^ Thus, effectively re‐educating M2‐like TAMs into the “eat me” phenotype plays a crucial role in tumor immunotherapy.^[^ ^35^ ^]^ We observed a striking increase in the phagocytosis of latex beads when Shisa3 was overexpressed in macrophages (Figure S8D, Supporting Information). GSEA‐Gene Ontology (GO) analysis showed Myeloid leukocyte activation, Antigen processing and presentation and Phagocytosis were the most enriched functional processes (Figure S8E, Supporting Information). We further explored the role of SHISA3 in macrophage phagocytosis of tumor cells, and flow cytometry results showed that Shisa3 overexpression in BMDMs attenuated tumor cells escape from macrophage phagocytosis (Figure 7D; Figure S8F, Supporting Information). Knockout Shisa3 in macrophages inhibits its phagocytic effect on tumor cells with or without LPS stimulation (Figure S8G, Supporting Information). These results indicated Shisa3 erexpression in BMDMs promotes phagocytosis. Next, we performed a subcutaneous tumor formation experiment in WT and Shisa3‐KO mice to evaluate the antitumor effects of this gene. Due to the low expression of Shisa3 at basal level (Figure S3A, Supporting Information), we found that there is no statistical significance between the two groups, although the tumor growth in Shisa3‐KO mice showed an accelerating trend (Figure S8H, Supporting Information). To further compare the antitumoral function of TAMs with or without Shisa3, TLR4 agonist MPLA (a derivative of LPS with lower inflammatory toxicity compared to LPS^[^ ^36^ ^]^ been approved for using as an adjuvant in human vaccines^[^ ^37^, ^38^ ^]^) was used to induce Shisa3 expression in BMDMs. The *Shisa3* and *PD‐L1* level in macrophages was significantly increased after MPLA stimulation (Figure S8I, Supporting Information), and Shisa3 KO inhibits the expression of iNos in macrophages induced by MPLA or LPS (Figure 7E). Moreover, intratumorally injected MPLA promoted the expression of Shisa3 and other proinflammatory cytokines in LLC tumors (Figure S8J,K, Supporting Information). In this model, combination therapy with MPLA and PD‐1 antibody suppressed tumor growth, which was largely abolished by Shisa3 knockout (Figure 7F,G). These results suggested that induced Shisa3 expression is indispensable for the antitumoral reprogramming of TAMs in cancer immunotherapy.

We further checked if the expression of SHISA3 in TAMs would affect disease progression in cancer patients. We first explored the clinical relevance of SHISA3 in the TIMER 2.0 database. The results showed that higher expression of SHISA3 correlated with a better prognosis for diverse cancers, including breast cancer, lung cancer, and pancreatic cancer (Figure 7H). We then performed multiplex immunofluorescence staining (mIHC) to accurately assess the expression of SHISA3 in TAMs and the proportion of CD8^+^ T cells in 81 patients with NSCLC. The patients were divided into high and low groups by the expression of SHISA3 in TME or the expression of SHISA3 in M1‐type TAMs. We found that high expression of SHISA3 or SHISA3 in M1‐type TAMs was associated with better overall survival in these patients (Figure 7I,J). High infiltration of CD8^+^ T cells is known to be relevant to prolonged overall survival of cancer patients.^[^ ^39^ ^]^ The mIHC results showed that the number of CD68^+^CD163^−^SHISA3^+^ antitumoral (M1) macrophages were proportionally correlated with the number of tumor‐infiltrating CD8^+^ T cells in these individuals (Figure 7K,L). In addition, CD8^+^ T cell levels were not associated with the bulk expression of SHISA3 (Figure S8L, Supporting Information). The percentages of CD68^+^CD163^−^SHISA3^+^ antitumoral macrophages showed no difference between the tumor and adjacent tissues (Figure S8M, Supporting Information). These data suggested that the high expression of SHISA3 in TAMs could restrain tumor progression by promoting antitumor immunity. Taken together, SHISA3 promises to be a therapeutic target for cancer immunotherapy.

## 3 Discussion

mAbs inhibit immune checkpoints and CAR‐T cell immunotherapy has shown promising therapeutic effects in some patients. However, these therapeutics are not entirely effective for certain tumors or patients as T cells are difficult to infiltrate into tumors and are largely repressed by immunosuppressive TME. Macrophages mediate the phagocytosis of tumor cells, as well as interact effectively with components of the innate and adaptive immune system.^[^ ^40^ ^]^ Given that TAMs are important components of TME, therapeutics based on macrophage polarization may overcome the limitations of other immunotherapies.^[^ ^41^ ^]^ For example, CAR‐Ms using recombinant adenoviral vectors could keep sustained pro‐inflammatory (M1) phenotype and resist the effects of immunosuppressive cytokines.^[^ ^17^ ^]^ Induced pluripotent stem cell‐derived macrophages (iMACs) with toll‐like receptor 4 intracellular toll/IL‐1R (TIR) domain‐containing CARs largely enhanced antitumor effect with both target engulfment capacity and antigen‐dependent M1 polarization as well as the capacity to modulate the tumor microenvironment.^[^ ^18^ ^]^ Cell derived TAMs targeted microparticles (MP) loaded with tumor antigen and TLR7 agonist polarizes M1 macrophage and boosts anti‐PD‐1 immunotherapy.^[^ ^42^ ^]^

In this study, we identified SHISA3 as a regulator of antitumoral macrophage polarization and reported a novel immunotherapy approach for tumors resistant to checkpoint blockade. We found that overexpression of SHISA3 in macrophages polarized them to the antitumoral type, thereby curbing tumor growth through enhancing phagocytosis of tumor cells and CD8^+^ T cell‐mediated anti‐tumor immunity. Mechanistically, DAMPs/PAMPs induced the expression of Shisa3, which interacted with Hspa8 to activate NF‐κB signaling mediated proinflammatory response and then increase TAMs repolarization in TME. Local delivery of Shisa3 mRNA or AAV‐Shisa3 transducted macrophages showed a superior effect when combined with PD‐1 blockade, suggesting that SHISA3 is a novel target for cancer immunotherapy.

Macrophage‐mediated phagocytosis is one of the most important functions of innate immune response. “Don't Eat Me” signals (CD47–SIRP,^[^ ^43^ ^]^ MHC I‐LILRB1,^[^ ^44^ ^]^ CD24‐Siglec‐10^[^ ^45^ ^]^) are the checkpoint for macrophages. The traditional strategies for activating macrophage phagocytosis include enhancing pro‐phagocytic signals by targeting “Don't Eat Me/Eat Me” signals. In this study, we use a different method to induce macrophage phagocytosis of tumors by reprograming TAMs through Shisa3 overexpression with AAV‐mediated transduction or NP‐mRNA transfection, which may show synergistic effects of the conventional strategies. Our findings indicate that Shisa3 not only promotes the repolarization of TAMs into an anti‐tumor phenotype but also enhances T‐cell activation, consequently converting “cold” tumors into “hot” tumors.

Multiple studies have shown that SHISA3 significantly inhibits tumor growth, reverses drug resistance, and inhibits tumor stem cell characteristics. SHISA3 attenuates Wnt/*β*‐catenin^[^ ^46^ ^]^ and MAPK signalings,^[^ ^24^ ^]^ and hypermethylation of SHISA3 DNA is a candidate biomarker.^[^ ^23^ ^]^ Studies have shown that SHISA3 DNA hypermethylation is a common phenomenon in many tumors. We accidentally found that the expression of Shisa3 could be greatly induced by LPS plus JAKi treatment. Although this phenomenon was observed in BMDMs, it is worthwhile to further explore the mechanism in cancer cells to restore their tumor suppressive function. Moreover, we showed that local administration of MPLA in combination with PD‐1 antibody can reprogram TAMs to an antitumoral phenotype in a Shisa3 dependent manner. We also showed that the higher expression of SHISA3 in TME or TAMs inhibits the progression of lung cancer patients and may promote CD8^+^ T cell infiltration. Therefore, we delivered Shisa3 mRNA to the tumor microenvironment by nanoparticles.^[^ ^47^ ^]^ This method has a dual function: on the one hand, after being engulfed by macrophages, Shisa3 can induce its inflammatory M1 polarization, improve the antigen presentation function of macrophages, and restore the infiltration and activity of T cells in tumors; on the other hand, Shisa3 can also be delivered to tumor cells, exerting its function as a tumor suppressor gene. Both of these aspects can limit the growth of tumors.

Collectively, this study provides proof‐of‐concept evidence that overexpression of the cancer suppressor gene SHISA3 in TAMs enhanced the innate and adaptive immune responses to tumor cells thus reshaping the immunosuppressive TME (Figure S9, Supporting Information), and would shed light on the development of new therapeutics for cancer immunotherapy.

## 4 Experimental Section

### Mice

The Shisa3‐deficient mice (Strain NO. T035425, C57BL/6J background) used in this study were generated by GemPharmatech (Nanjing, China) using CRISPR/Cas9 gene targeting technology. C57BL/6 wide‐type mice were obtained from Guangdong Experimental Animal Center (License Number: SCXK 2018‐0002). All experimental mice were bred and maintained under specific pathogen‐free conditions at Sun Yat‐sen University (Guangzhou, China). Six‐ to 12‐week‐old mice of both sexes were used for the experiments. Wild‐type C57BL/6 mice were used as controls. All animal experiments were approved by the Institutional Animal Care and Use Committee of the Sun Yat‐sen University Cancer Center (SYSUCC; Guangzhou, China).

### Cell Culturing and Human Tissues

LLC, 4T1 cells, 293T cells, J774A.1, THP1, RAW 264.7 were obtained from American Type Culture Collection (ATCC) and cultured with a DMEM medium supplemented with 10% fetal bovine serum (Thermo Fisher Scientific, Gibco, Birmingham, MI, USA) and antibiotic (streptomycin‐penicillin) at 37 °C with 5% CO_2_. 81 cases of Paraffin‐embedded specimens from lung cancer tissue were obtained from Sun Yat‐Sen University Cancer Center.

### Differentiation and Polarization of BMDMs

Macrophages were differentiated from the bone marrow of wild‐type (WT) C57BL/6 mice and *Shisa3*‐KO mice. All bone marrow cells were flushed out and filtered through a 70‐µm cell strainer. After centrifugation, red blood cells were lysed. The resultant bone marrow cells were resuspended in RPMI 1640 (Gibco) supplemented with 10% FBS (Gibco), 1% penicillin/streptomycin (Gibco), and 50 µm 2‐mercaptoethanol (Sigma) in the presence of 20 ng mL^−1^ macrophage colony‐stimulating factor (M‐CSF; PeproTech) for 7 days. BMDMs were stimulated with 100 ng mL^−1^ LPS (Sigma) with or without 20 ng mL^−1^ IFN‐*γ* (PeproTech) or 20 ng mL^−1^ Il‐4 (PeproTech) separately for 24 h to obtain antitumoral‐ or protumoral‐type macrophages, respectively.

### Tumor Models and Treatments

1 × 10^6^ LLC tumor cells were implanted s.c. into the flanks of mice. For in vivo mRNA administration, the in vivo‐jetPEI (Polyplus transfection) was used for the injection of mouse tumors. Briefly, 14 µl of PEI reagent + 7 µg of Shisa3 mRNA was used per mouse; control mice were treated with 14 µL of PEI reagent + 7 µg of GFP mRNA. Treatment was started 15 days later when xenografts reached 100–200 mm^3^. Both groups of mice received the intratumoral injections. Tumor volume and body weight were recorded every two days and the formula of volume calculation is that V = length × width2 × 0.5. Tumor‐bearing mice were sacrificed if the volume reached 2000 mm^3^. For cotransferring with macrophages, LLC (5 × 10^5^) were mixed with an equal number of BMDMs and implanted s.c. into the flanks of wild‐type C57BL/6 mice. For combination therapy, a similar protocol was performed 200 µg anti‐PD‐1 antibody per mouse was injected intraperitoneally the following day after mRNA administration.

### AAV Production and Purification

All AAV vectors were produced in HEK293 cells via the triple plasmid transient transfection method as described previously. For small‐scale preps, HEK293 cells were seeded in 10‐cm dishes and grown to 80% confluence in Dulbecco's modified Eagle's medium (DMEM) containing 10% fetal bovine serum (FBS) (Gibco, 26140079) and 1% PenStrep (Thermo Fisher Scientific, 15140122). Cells were then triple transfected with the vector pscAAV‐CAG‐GFP (Addgene, 83279) or pscAAV‐CAG‐Shisa3, AAV1 Rep/Cap (Addgene, 112862), and Ad helper plasmid (pAddelta F6 from Addgene, 112867) at a ratio of 1:1.2:2 (3.75:4.5:7.5 µg per 10‐cm dish) using PEI MW40000, pH 7.1 (Yeasen Biotechnology, 40816ES03 at a ratio 4:1 of PEI/total DNA. Cells were harvested 3 days post transfection by scraping cells off the plate in their conditioned medium and lysing cells through 3× freeze‐thaw cycles between 37 and −180 °C. Preps from three replicate plates were then pooled, incubated with 25 U mL^−1^ of benzonase (Millipore Sigma, E8263‐25KU) at 37 °C for 1 h to remove plasmid and cell DNA, centrifuged at 4 °C and 4000 × g for 30 min, and the supernatant filtered through a 0.22‐µm polyethersulfone (PES) bottle‐top filter (Corning, 431097). The filtered lysate was Purificated by iodixanol gradient ultracentrifugation. For AAV collection, the fractions obtained from the 40% phase were analyzed by measuring absorbance at 20‐fold dilution at 340 nm to identify the main contaminating protein peak, as previously described. For ultrafiltration/concentrated AAV, 0.001% Pluronic F68 + 200 mm NaCl PBS was added to the pool to reach a total volume of 15 mL, using Amicon Ultra‐15 centrifugal filter units (MWCO, 100 kDa; Merck Millipore). After concentration to a minimum of 500 µL, the product was aliquoted and stored at −80 °C.

### Flow Cytometry

Single‐cell suspensions were prepared from the tumor tissues of mice, as described previously. Tumor tissues were cut into small pieces and washed with PBS containing 2% FBS. The tumors were digested in 15 mL RPMI supplemented with 2% FBS, 50 U mL^−1^ Collagenase Type IV (Invitrogen, California, USA), 20 U mL^−1^ DNase (Roche, Indianapolis, IN) and incubated at 37 °C for 30 min to 1 h while gently shaking. Digested tumors were then filtered through a 70 µm strainer after washed three times with PBS. Spleens were mechanically dissociated with gentleMACS dissociator in RPMI‐1640 medium supplemented with 2% FBS. Dissociated spleens were passed through a 70 µm strainer and washed three times with PBS. Red blood cells were lysed for 3–5 min with ACK lysis buffer and then washed with PBS containing 2% FBS. Single cells were stained with the appropriate antibodies to surface markers at 4 °C for 30 min in the dark. Intracellular CD206 and intracellular cytokine staining was done using the BD Bioscience Cytofix/CytopermTM solution according to the manufacturer's protocols. The following fluorescent dye‐labeled antibodies purchased from BD Biosciences, Biolegend or Invitrogen were used in this study: CD3ε (145‐2C11), CD4 (GK1.5), CD8 (KT15), CD11b (M1/70), CD45 (30‐F11), CD44 (IM7), F4/80 (BM8), PD‐L1 (MIH7), Gr‐1 (RB6‐8C5), IFN*γ* (XMG1.2), iNOS (CXNFT), CD206 (C068C2), CD19 (1D3), CD107a (1D4B). For flow cytometry analysis, single cells from tumors were gated on CD45^+^ cells and were further analyzed using the indicated markers to characterize the infiltration and activation of macrophages and T cells. All flow cytometric data were collected on BD Fortessa X20 (BD Biosciences, San Jose, CA) and performed using FlowJo analysis software v10.4.

### Mass Spectrometry and Coimmunoprecipitation

Cells were prepared by washing with cold PBS and then lysed with 1× lysis buffer (Cell Signaling Technology) and incubated on ice for 30 min. Supernatants were collected and immunoprecipitated with the indicated antibodies for 1 h at 4 °C, recovered by adding protein A/G Sepharose Beads (Santa Cruz Biotechnology, CA, USA, #sc‐2002) overnight. After incubation, beads were washed with wash buffer. The eluted protein complexes were denatured and then subjected to mass spectrometry. Immunoblot assays were performed with specific antibodies to identify the proteins interacting with SHISA3. The following antibodies were used for Co‐IP or immunoblot assay: SHISA3 (Invitrogen, #PA5‐34527), Flag (Sigma, St. Louis, USA, #F1804), and HA (Sigma, St. Louis, USA, #H9658).

### Dual‐Luciferase Reporter Assay

Cells were transfected with plasmids encoding NF‐κB or IL‐6‐ΔNF‐κB luciferase reporter together with pRL‐TK and the plasmids encoding SHISA3. Cells were collected and lysed 24 h post‐transfection. Subsequently, the luciferase activities were measured using a Dual‐luciferase Reporter Assay System (Promega, Madison, USA, #E1910). Normalization of data by the ratio of firefly luciferase activity to renal luciferase activity. Each group was measured in triplicate.

### RNAi

In this study, the siRNA sequences were designed as follows:

si‐Shisa3‐1, 5′‐TGTCCAGATTTCAGTTCCA‐3′; si‐Shisa3‐2, 5′‐CTTCATCGCCTTCATCATT‐3′. si‐Socs1‐1, 5′‐GCAGCCGACAAUGCGAUCUTT‐3′; si‐Socs1‐2, 5′‐GGAACUGCUUCUUCGCGCUTT‐3′. si‐Hspa8‐1, 5′‐GCUCGAUUUGAGGAGUUGAAUTT‐3′; si‐Hspa8‐2, 5′‐CCCUAUCAUUACCAAGCUGUATT‐3′. All the siRNA oligonucleotides containing 3′dTdT overhanging sequences were chemically synthesized in RIBOBIO (Guangzhou, China) and transfected into cells using Lipofectamine RNAiMAX Transfection Reagent (Thermo Fisher Scientific) according to the manufacturer's instructions. A negative control nucleotide (si‐NC) was also purchased from RIBOBIO (Guangzhou, China).

### RNA Extraction and Quantitative Real‐Time PCR

Total RNA from cells was extracted with Trizol reagent (Magen, R4801) according to the manufacturer's instructions. The quantity and quality of RNA were measured by Nanodrop. cDNA was synthesized using the HiScript II Q RT SuperMix for qPCR (Vazyme, R333‐01). Quantitative real‐time PCR was performed in a 384‐well format on a Real‐Time PCR Detection System (Bio‐Rad) using Hieff qPCR SYBR Green Master Mix (YEASEN, 11201ES08). Relative quantitation was performed with the 2(‐ΔΔCT) method using 18S (for human cells) or Actb (*β*‐Actin, for mouse cells) for normalization. The Specific qRT‐PCR primers are listed in Table S1 (Supporting Information).

### ELISA of Cytokines

IL‐1*β* secreted by BMDMs in the culture medium was detected by using an ELISA Kit (Elabscience, E‐HSEL‐M0001) according to the manufacturer's guidelines. Absorbance at 450 nm was measured using a multi‐wavelength measurement system (TECAN).

### Phagocytic Assay

For the beads‐based phagocytic assay, BMDMs were cultured with 4 µL of fluorescent blue–labeled latex beads (size 2 µm; Sigma; #L0280) for 1 h. Cells that phagocytized latex beads were analyzed by FACS and CD11b^+^ macrophages that express blue fluorescence were counted. For phagocytic of tumor cells, tumor cells (Raji, K562, MC‐38) were stained with CellTrace Far Red (Invitrogen, # C34572) for 30 min at 37 °C, then, BMDMs were co‐cultured with stained tumor cells for 2 h. BMDMs that phagocytized tumor cells were analyzed by FACS.

### mRNA Synthesis

In vitro transcription of GFP‐mRNA or Shisa3‐mRNA was synthesized using T7 polymerase‐conducted transcription. Briefly, Shisa3‐plasmid or GFP‐plasmid carrying the open reading frame (ORF) with a T7 promoter was digested by restriction enzymes (HindIII‐5′UTR‐BamHI‐GENE‐EcoRI‐3′UTR‐XbaI), Xba‐I site was used for plasmid linearization for in vitro transcription, followed by DNA purification. Next, chemically modified 5′ and 3′ untranslated regions (UTRs) were added to the flanking of the Shisa3 or GFP open reading frame. A CleanCap was added to the 5′ end and a length of 120 nt polyadenosine (poly‐A) tail was added to the 3′ end to effectively facilitate the production of in vitro transcription transcripts. Then the transcripts were treated with DNase and Phosphatase, followed by purification according to the manufacturer's instructions. The mRNA quality and concentration identification by gel electrophoresis and Nanodrop. The final mRNA products were diluted in nuclease‐free water and stored at −80 °C.

### ChIP Assays

ChIP assays were performed using a ChIP Assay Kit (Beyotime, #P2078) according to the manufacturer's instructions. Briefly, 1.5 × 10^7^ BMDMs were seeded on a 10‐cm dish. The next day, cells were stimulated for 2 h with or without LPS. Cells were then cross‐linked for 10 min at room temperature in 1% formaldehyde PBS (Thermo Fischer, # 28906) and quenched with 0.125 m glycine for 10 min. Cells were washed three times with ice‐cold PBS supplemented with 1 mm PMSF. Cells were harvested and centrifuged for 5 min at 1000 × g at 4 °C. Cell precipitation was resuspended with 0.2 mL SDS Lysis Buffer containing 1 mm PMSF. After cell lysis, Samples were sheared using Bioruptor Pico (Diagenode) with 30s on/45s off for eight cycles. Chromatin concentration was measured by NanoDrop. Sonicated samples were then used for immunoprecipitation with 10 µL of anti‐phospho‐NF‐κB p65 (Ser536; #3033, Cell Signaling Technology) or corresponding normal rabbit IgG (#2729, Cell Signaling Technology) and rotated at 4 °C overnight. After reversal of cross‐linking, the DNA immunoprecipitated was purified and concentrated, and tested by qPCR using primers listed in Supplementary Table.

### RNA‐seq

A total amount of 1–3 µg RNA per sample was used as input material for the RNA sample preparations. Sequencing libraries were generated using VAHTS Universal V6 RNA‐seq Library Prep Kit for Illumina (NR604‐01/02) following the manufacturer's recommendations and index codes were added to attribute sequences to each sample. RNA concentration of the library was measured using Qubit RNA Assay Kit in Qubit 3.0 to preliminary quantify and then dilute to 1 ng µL^−1^. Insert size was assessed using the Agilent Bioanalyzer 2100 system (Agilent Technologies, CA, USA). After the insert size met the expectation, the Bio‐RAD CFX 96 fluorescence quantitative PCR instrument was used to accurately quantify the library effective concentration (Library effective concentration > 10 nm), and the reagent used was Bio‐RAD KIT iQ SYBR GRN. The clustering of the index‐coded samples was performed on a cBot cluster generation system using HiSeq PE Cluster Kit v4‐cBot‐HS (Illumina) according to the manufacturer's instructions. After cluster generation, the libraries were sequenced on an Illumina platform and 150 bp paired‐end reads were generated. The cluster generation and sequencing were performed on the Novaseq 6000 S4 platform, using NovaSeq 6000 S4 Reagent kit V1.5.

### Multiplexed Immunohistochemical Staining

Multiplex staining of paraffin‐embedded liver tissues was performed using a Panovue 7‐plex IHC kit (Panovue, # 0004100100,). CD68 (1:100, # TA802955, ZSGB‐bio), CD8(1:100, # TA802376, ZSGB‐bio), CD163(1:100, # TA506381, ZSGB‐bio), SHISA3 (Invitrogen, #PA5‐34527) antibodies were sequentially applied, followed by horseradish peroxidase‐conjugated secondary antibody incubation and tyramide signal amplification (TSA). After all antigens above have been labeled, nuclei were stained with 4′‐6′‐diamidino‐2‐phenylindole (DAPI, Sigma‐Aldrich, Missouri, USA, Cat# D9542).

### Digital Image Analysis for Cell Densities

The stained slides were scanned to obtain multispectral images using the Vectra Polaris Automated Quantitative Pathology Imaging System (Akoya Biosciences, Menlo Park, CA) and analyzed using Phenochart image analysis software (version 1.2.0, Akoya Biosciences). Quantification of pathologic lesions was performed with HALO software (HALO V2.0, Indica Labs, Corrales, NM).

### Analysis of Data from TCGA and TIMER 2.0

Gene expression and clinical data for TCGA were downloaded for analysis by logging into TCGA, selecting Access TCGA Data and entering the database. Survival analyses were performed using the “survival” R package (version 3.2‐13) and Kaplan–Meier methods with a log‐rank test. TIMER2.0 (http://timer.cistrome.org/) was applied to analyze the association between immune infiltrates and SHISA3 expression, TIMER2.0 utilizes an R package that integrates six state‐of‐the‐art algorithms, these algorithms have been systematically benchmarked. For M1 macrophage and CD8^+^ T cell infiltration, QUANTISEQ algorithm was used for all cancer types. For MDSC infiltration, TIDE algorithm was used for all cancer types. The results showed the purity‐adjusted spearman's rho across various cancer types. The correlation between SHISA3 and immune check point genes data was downloaded from TIMER2.0. Specifically, select the Exploration component and enter the Gene_Corr module, then download the data and draw correlation heatmaps through the R language “corrplot” package.

### Statistical and Reproducibility

All experiments were performed at least three times. Data were presented by descriptive statistics such as mean with s.d. Statistical analyses were performed using the GraphPad Prism Software v.8.0 and a Student's t‐test or paired t‐test was used to compare two independent or matched conditions/groups, and one‐way analysis of variance (ANOVA) was used to identify the significance among multiple groups except when stated otherwise. Significant P values were indicated by asterisks in the figures as follows: ^∗^ *p* < 0.05, ^∗∗^ *p* < 0.01, ^∗∗∗^ *p* < 0.001, ^****^ *p* < 0.0001, and ns = non‐significant. The survival curve was performed via the Log‐rank (Mantel‐Cox) test and a p‐value of <0.05 was considered statistically significant.

### Ethics Approval and Consent to Participate

The study with human clinical samples was approved by the Sun Yat‐Sen University Cancer Center institutional review board (approval number: B2023‐679‐01). All animal studies were carried out according to the National Institute of Health Guide for the Care and Use of Laboratory Animals with the approval of Sun Yat‐Sen University Cancer Center Institutional Animal Care and Use Committee (approval number: SYSU‐IACUC‐2022‐001827).

### Availability of Data and Materials

Raw data of the RNA‐seq that support the findings of this study have also been deposited at the GEO (GSE255711).

## Conflict of Interest

The authors declare no conflict of interest.

## Author Contributions

S.Z., B.Y., and C.S. contributed equally to this work. S.Z. performed most of the experiments and analyses. B.X. and B.Z. performed mRNA synthesis. S.Z., C.Y. and J.W. performed the mouse experiments. S.Z., C.S., Y.L. and J.B. performed RNA‐seq and bioinformatic analyses. Q.Z. and Y.M provided technical assistance. S.C. conceived the study. S.C. and S.Z. wrote the manuscript.

## Supporting information

Supporting Information

## Data Availability

Raw data that support the findings of this study has been deposited in the Research Data Deposit database (http://www.researchdata.org.cn) with the Approval Number RDDB2024396758.
