# Supplementary material for: SHISA3 Reprograms Tumor‐Associated Macrophages Toward an Antitumoral Phenotype and Enhances Cancer Immunotherapy
Source: Adv Sci (Weinh). 2024 Jul 25;11(36):2403019. doi: 10.1002/advs.202403019 (PMC11423144; doi:10.1002/advs.202403019)
Supplement: Supplementary file 1 — Supporting Information [file ADVS-11-2403019-s001.pdf]

ADVANCED SCIENCE
Open Access

# Supporting Information

for *Adv. Sci.*, DOI 10.1002/advs.202403019

SHISA3 Reprograms Tumor-Associated Macrophages Toward an Antitumoral Phenotype and Enhances Cancer Immunotherapy

*Shimeng Zhang, Bingbing Yu, Chunjie Sheng, Chen Yao, Yang Liu, Jing Wang, Qi Zeng, Yizhi Mao, Jinxin Bei, Bin Zhu and Shuai Chen**

**Supporting Information**

# SHISA3 reprograms tumor-associated macrophages toward an antitumoral phenotype and enhances cancer immunotherapy

Shimeng Zhang, Bingbing Yu, Chunjie Sheng, Chen Yao, Yang Li, Jing Wang, Qi Zeng, Yizhi Mao, Jinxin Bei, Bin Zhu, Shuai Chen

These supporting information include:

1) Figures S1 to S9

2) One supplementary table

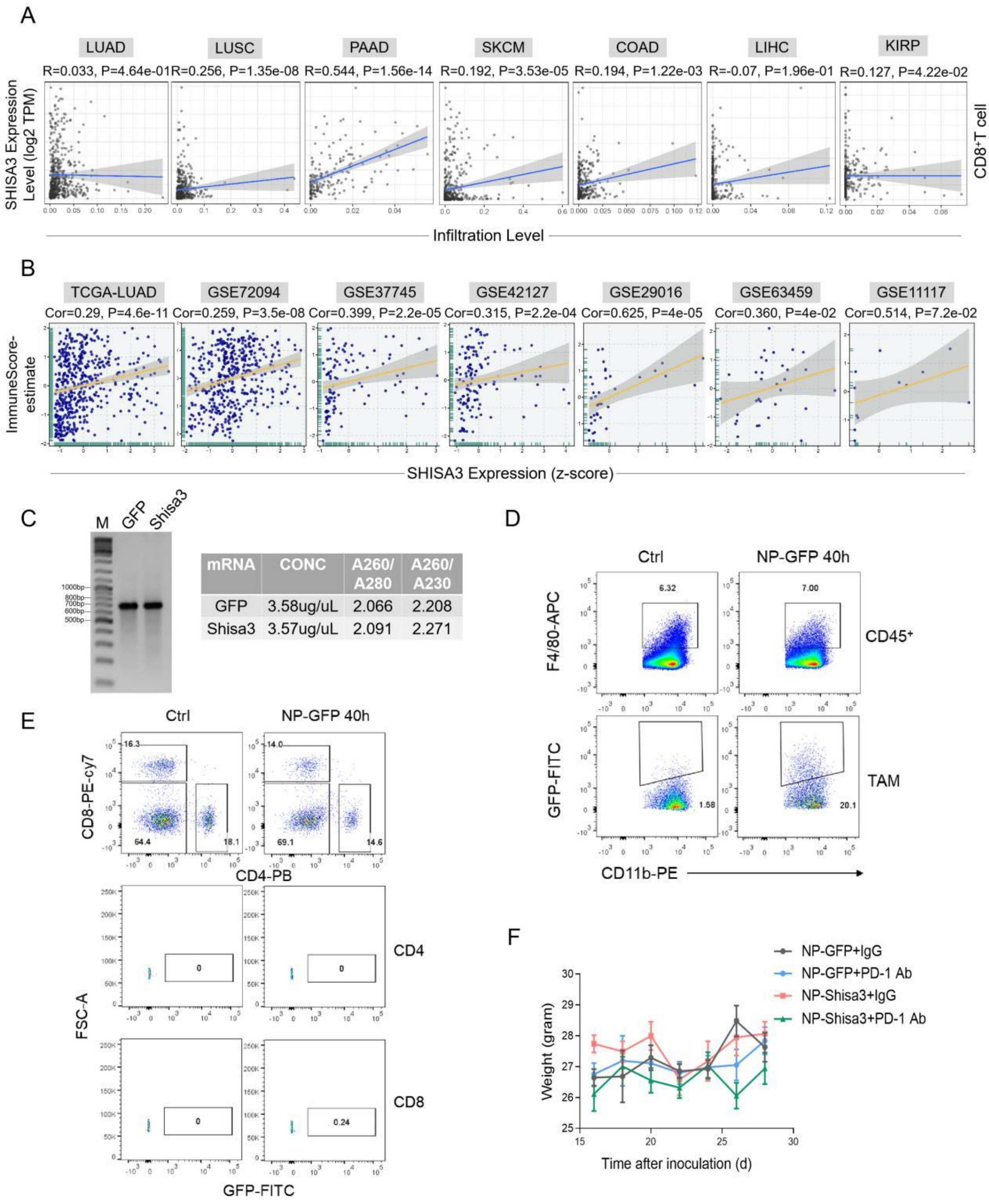


| mRNA | CONC | A260/ A280 | A260/ A230 |
|---|---|---|---|
| GFP | 3.58ug/uL | 2.066 | 2.208 |
| Shisa3 | 3.57ug/uL | 2.091 | 2.271 |

**Figure S1. The clinical significance of SHISA3 in cancers and local delivery of Shisa3 mRNA by nanoparticles. A)** Correlation between SHISA3 expression and and CD8$^+$ T cells infiltration by TIMER 2.0 website (http://timer.cistrome.org/). **B**) Scatter plots showing a positive Spearman's correlation between SHISA3 expression and immune scores calculated by the ESTIMATE algorithm from BEST website (https://rookieutopia.com/app_direct/BEST/). **C)** mRNA quality and concentration identification by gel electrophoresis and Nanodrop. **D**, **E)** Flow cytometry assay of GFP$^+$ TAMs (**D**) or GFP$^+$ T cells (**E**) of LLC tumors intratumoral injected with NP-GFP for 40h. **F)** Body weight of mice during treatment. Data are presented as mean ± SD and were analyzed by one-way ANOVA (**F**). Data are representative of at least two independent experiments.

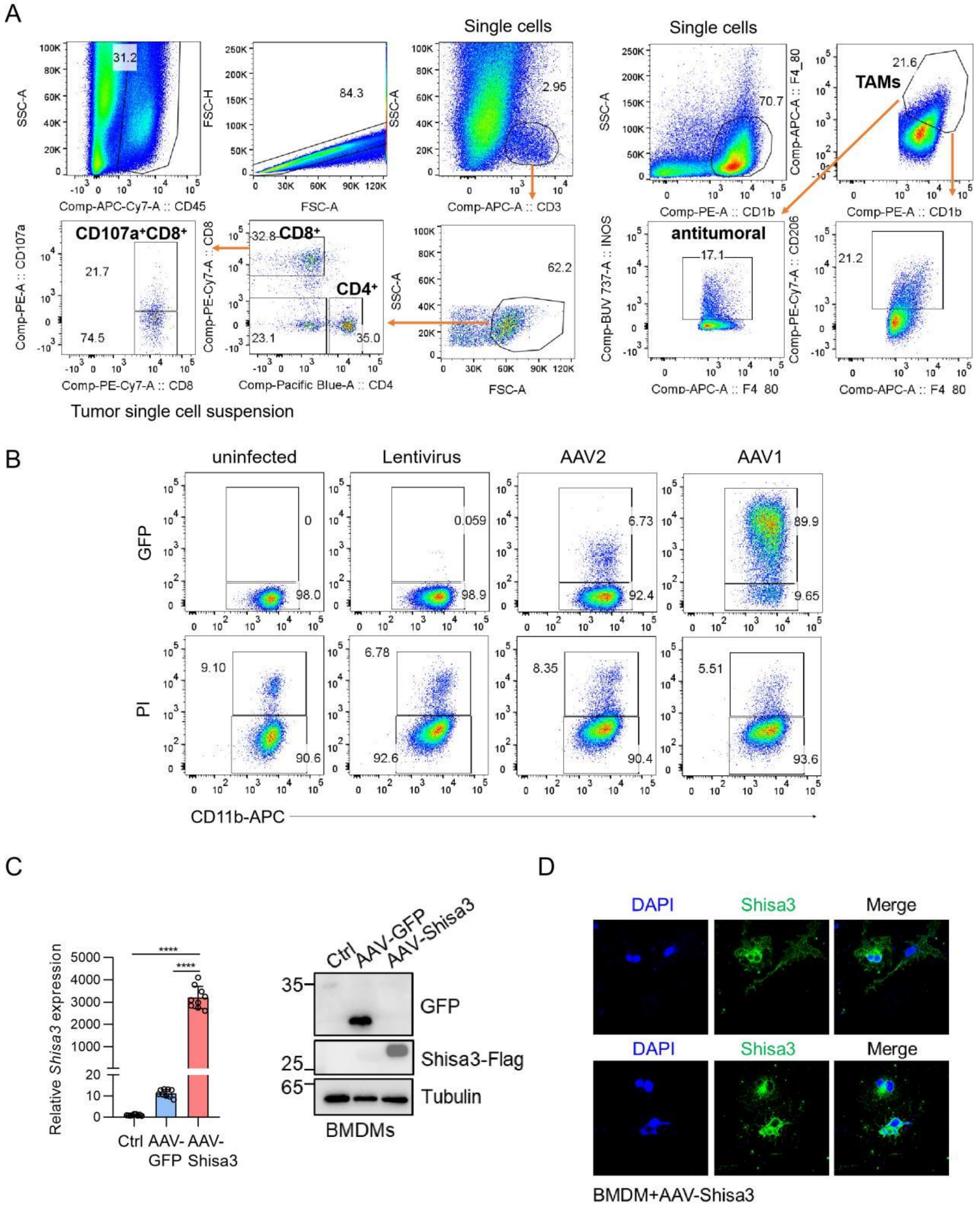


**Figure S2. Gating strategy of FACS analyses and overexpression of SHISA3 in BMDMs by AAV infection.** **A)** gating strategy for TAMs and T cells of tumor single cell suspension. FSC: forward scatter, SSC: side scatter. **B)** Representative dot-plots of GFP and PI staining in gated CD11b$^+$ cells after lentivirus or AAV infection for 48 hours. **C**) Shisa3 mRNA (left) and protein (right) expression in BMDMs after AAV-GFP or AAV-Shisa3 infected for 48 hours. **D)** Immunofluorescence staining showing the cellular location and expression efficiency of Flag-tagged Shisa3 (green) in BMDMs. Data are presented as mean ± SD and were analyzed by one-way ANOVA (**C**). ****$p < 0.0001$. Data are representative of at least three independent experiments.

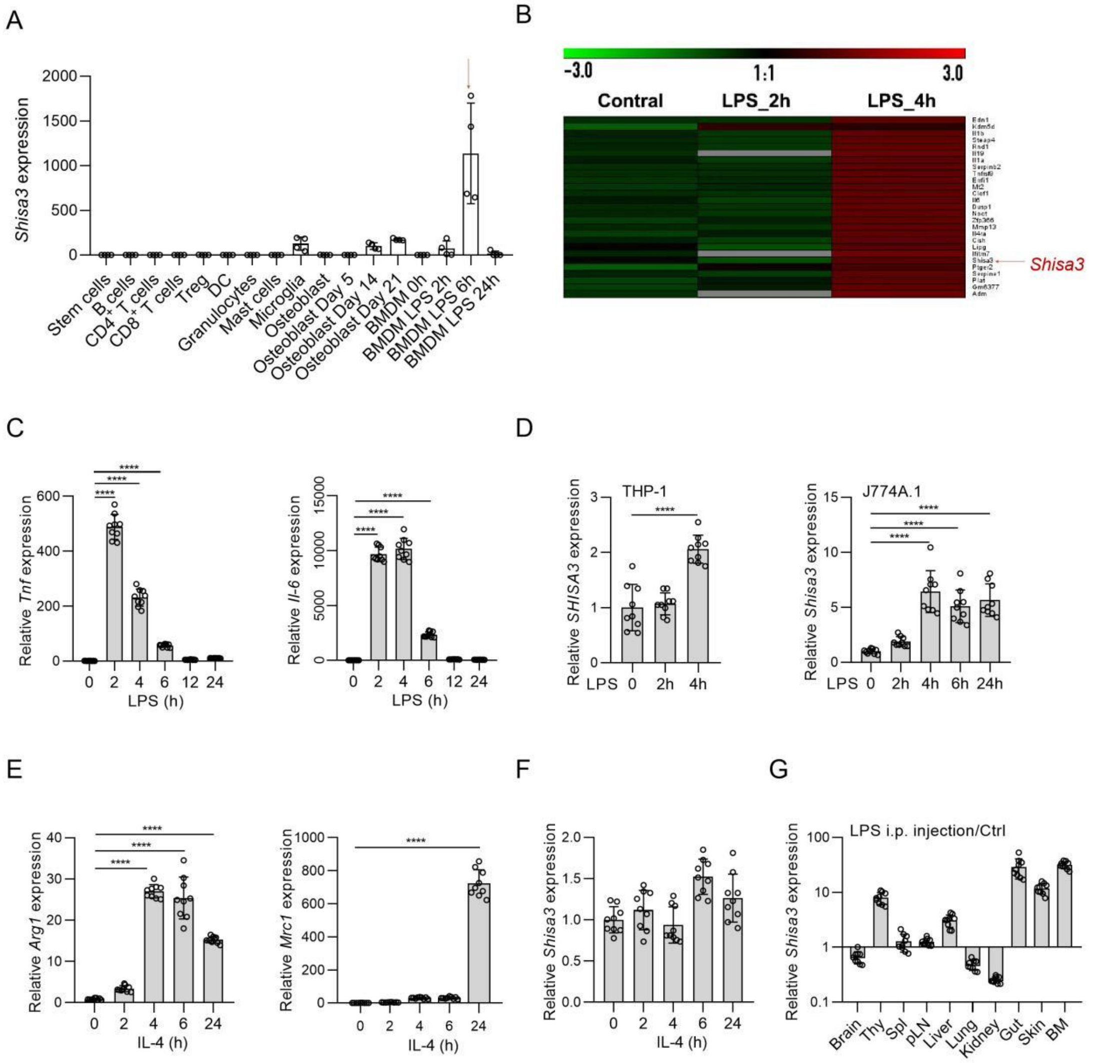


**Figure S3. SHISA3 is an inflammation-related gene. A)** Analysis of *Shisa3* expression in various cells of mice using the BioGPS database (http://biogps.org/). **B)** Heatmap depicting expression patterns of the indicated genes across Mo and LPS-stimulated M1 type macrophages. **C**) qRT-PCR of *Tnf* and *Il-6* expression in BMDMs after LPS stimulation. **D**) qRT-PCR of SHISA3 expression in J774A.1 cell or THP-1 cells after LPS stimulation. **E, F)** qRT-PCR of *Arg1* (**E** left) and *Mrc1* (**E** right) and *Shisa*3 expression (**F**) in BMDMs after Il-4 stimulation. **G**) qRT-PCR of *Shisa3* expression of organs in LPS induced acute inflammation mouse model. Data are presented as mean ± SD and were analyzed by one-way ANOVA (**C** to **G**). ****p < 0.0001. Data are representative of at least three independent experiments.

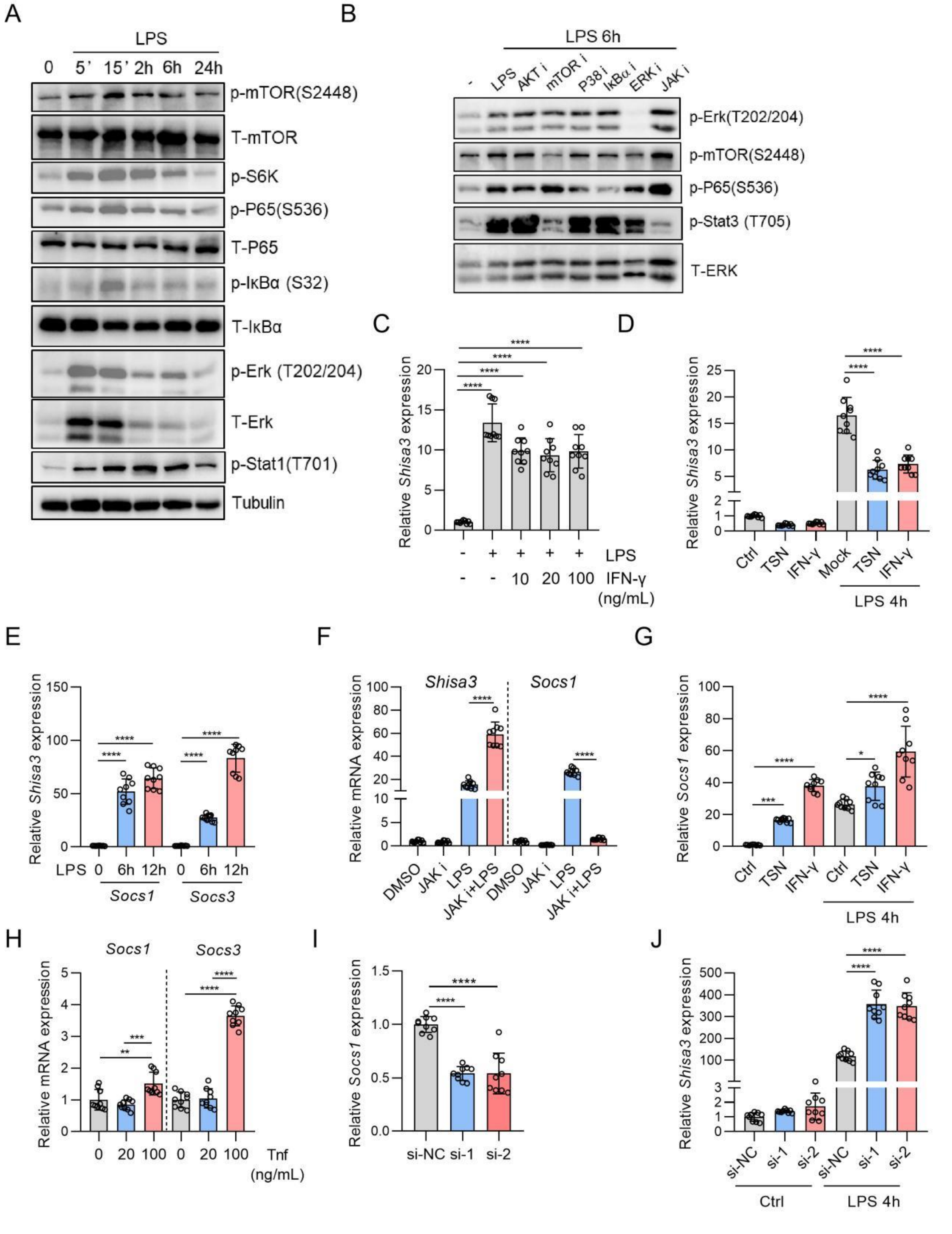


**Figure S4. SHISA3 expression is regulated by LPS/TLR4/NF-κB and JAK-STAT signaling in macrophages. A**) Western blot analysis of NF-κB, MAPKs, mTOR, and JAK-STAT activation in BMDMs after LPS stimulation at the indicated time point. **B)** Phosphorylation of indicated protein in BMDMs was examined by western blotting after LPS stimulated with or without the indicated inhibitors for 6h. **C)** qRT-PCR of *Shisa3* expression in BMDMs treated with LPS and the indicated concentration of IFN-γ for 4 hours. **D**) qRT-PCR of *Shisa3* expression in BMDMs treated with LPS ± IFN-γ (or OVA Peptide stimulated OT-1 mouse CD8$^+$ T cell supernatant (TSN)) for 4 hours. **E**) qRT-PCR of *Socs1* and *Socs3* expression in BMDMs after LPS stimulation. **F)** qRT-PCR of *Shisa3* and *Socs1* expression in BMDMs treated with Ruxolitinib with or without LPS. **G**) qRT-PCR of

*Socs1* expression in BMDMs treated with LPS±IFN-γ or TSN for 4 hours. **H)** qRT-PCR of *Socs1* and *Socs3* expression in BMDMs stimulated with TNF at the indicated concentration for 12h. **I)** qRT-PCR of the knockdown efficiency of *Socs1* in BMDMs transfected with Socs1 siRNA (si-Socs1-1/-2) or control siRNA (si-NC). **J**) qRT-PCR of *Shisa3* expression in BMDMs transfected with Socs1 siRNA (si-Socs1-1/-2) or negative control siRNA (si-NC), followed by LPS stimulation for 4 hours. Data are presented as mean ± SD and were analyzed by one-way ANOVA (**C** to **J**). *$p < 0.05$, **$p < 0.01$, ***$p < 0.001$, ****$p < 0.0001$. Data are representative of at least three independent experiments.

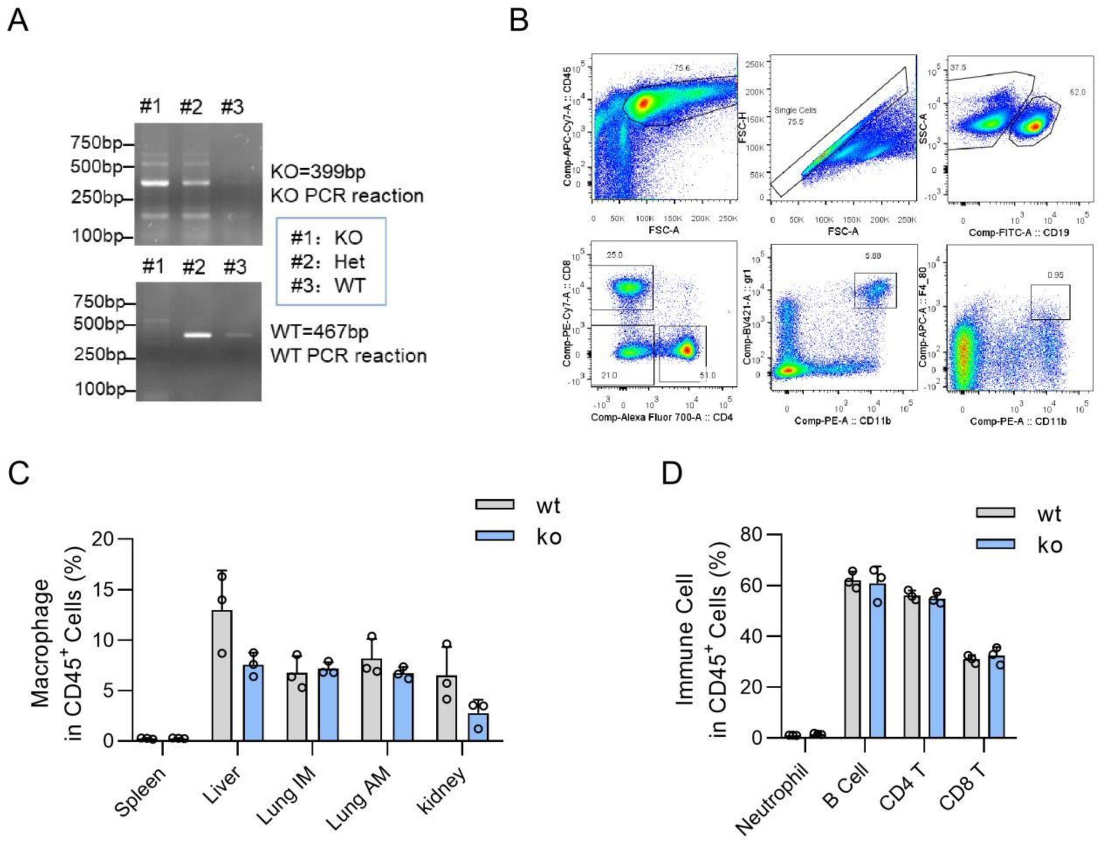


**Figure S5. SHISA3 deficiency does not affect the composition of immune cells of mice. A)** Genotype identification of *Shisa3*-KO mice by PCR and gel electrophoresis. **B-D)** Gating strategy (**B**), percentages of macrophages in different organs (**C**) and immune cells ($CD19^+$ B cells, $CD11b^+$ $F4/80^+$ macrophages, $CD11b^+$ $gr1^+$ MDSCs, $CD4^+$ T cells and $CD8^+$ T cells) (**D**) in the spleen of WT and *Shisa3*-KO mice.

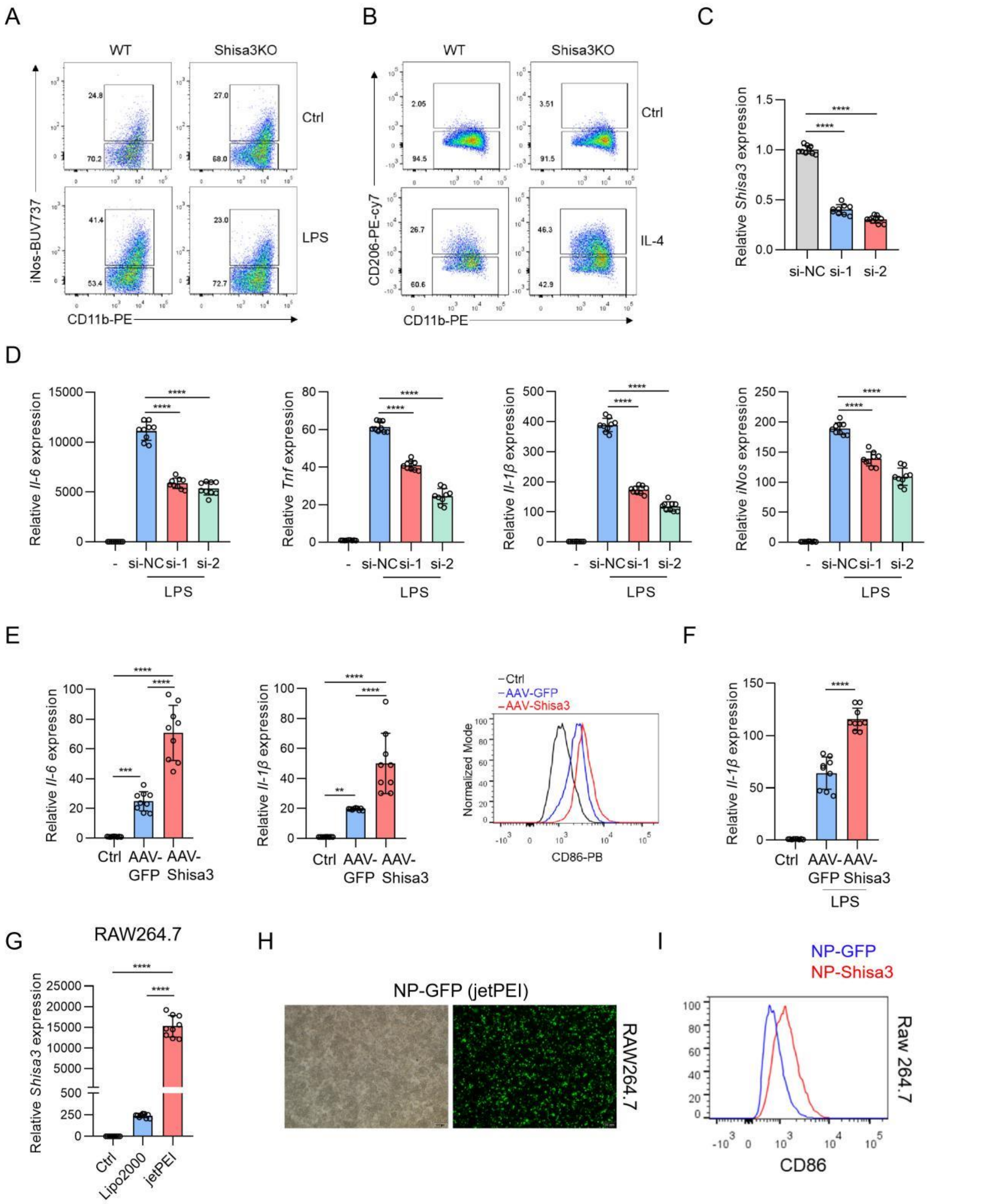


**Figure S6. SHISA3 drives M1 macrophage polarization. A)** Representative dot-plots of CD11b and iNos staining in gated CD11b$^+$ live cells from WT and *Shisa3*-KO BMDMs after LPS stimulation for 24 hours. **B)** Representative dot-plots of CD11b and CD206 staining in gated CD11b$^+$ live cells from WT and *Shisa3*-KO BMDMs after IL-4 stimulation for 24 hours. **C)** qRT-PCR of *Shisa3* knockdown efficiency in BMDMs transfected with Shisa3 siRNA (si-Shisa3-1/-2) or control siRNA (si-NC). **D)** qRT-PCR of the M1 markers in BMDMs transfected with Shisa3 siRNA (si-Shisa3-1/-2) or control siRNA (si-NC) after LPS stimulation for 4 hours. **E**) qRT-PCR of *Il-6* and *Il-1*β mRNA expression in uninfected, AAV-GFP or AAV-Shisa3 infected BMDMs. **F**) qRT-PCR of *Il-1*β mRNA expression in uninfected, AAV-GFP or AAV-Shisa3 infected BMDMs after LPS stimulation. **G)** qRT-PCR of *Shisa3* expression in RAW264.7 after mRNA transfection by

Lipo2000 or in vivo-jetPEI for 36h. **H)** Fluorescent images of GFP in RAW264.7 after transfected with GFP mRNA by in vivo-jetPEI for 36h. **I)** Flow cytometry assay of CD86 level in NP-Shisa3 or NP-GFP transfected RAW264.7. Data are presented as mean ± SD and were analyzed by one-way ANOVA (**C-G**). ****p < 0.0001. Data are representative of at least three independent experiments.

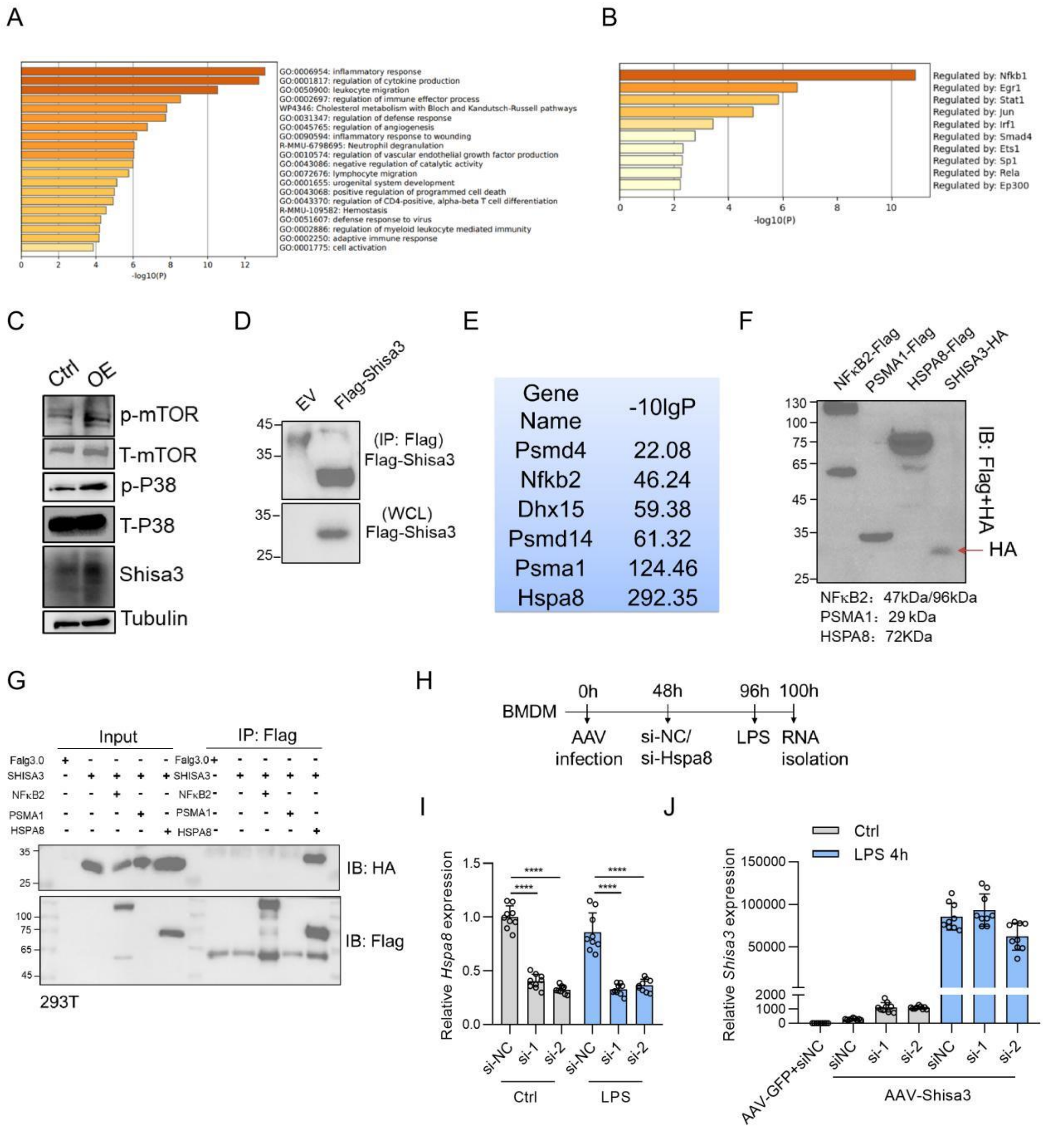


**Figure S7. Hspa8 mediates Shisa3-induced NF-κB activation in M1 polarization. A**, **B**) Gene Ontology (GO) category analysis (**A**) and Heatmap GO analysis (**B**) of RNA-seq data of AAV-Shisa3 and AAV-GFP transduced BMDMs by using Metascape website (http://metascape.org). **C)** Western blot analysis of AAV-GFP (Ctrl) and AAV-Shisa3 (OE) transduced BMDMs. **D)** Flag-tagged Shisa3 expressed by BMDMs were immunoprecipitated using anti-FLAG antibody. **E)** Protein interacted with Shisa3 screened from mass spectrometry results. **F)** Western blot analysis of enforced expression of indicated protein in HEK293T cells 24 h after transfection. **G)** Western blot analysis of interactions between SHISA3 and indicated protein by coimmunoprecipitation with anti-HA antibody in HEK293T cells. **H)** Schematic showing the treatment plan in BMDMs. **I, J)** qRT-PCR of *Hspa8* or *Shisa3* mRNA expression in BMDMs treatment according to (**H**). Data are presented as mean ± SD and were analyzed by one-way ANOVA (**I, J**). ****$p < 0.0001$. Data are representative of at least three independent experiments.

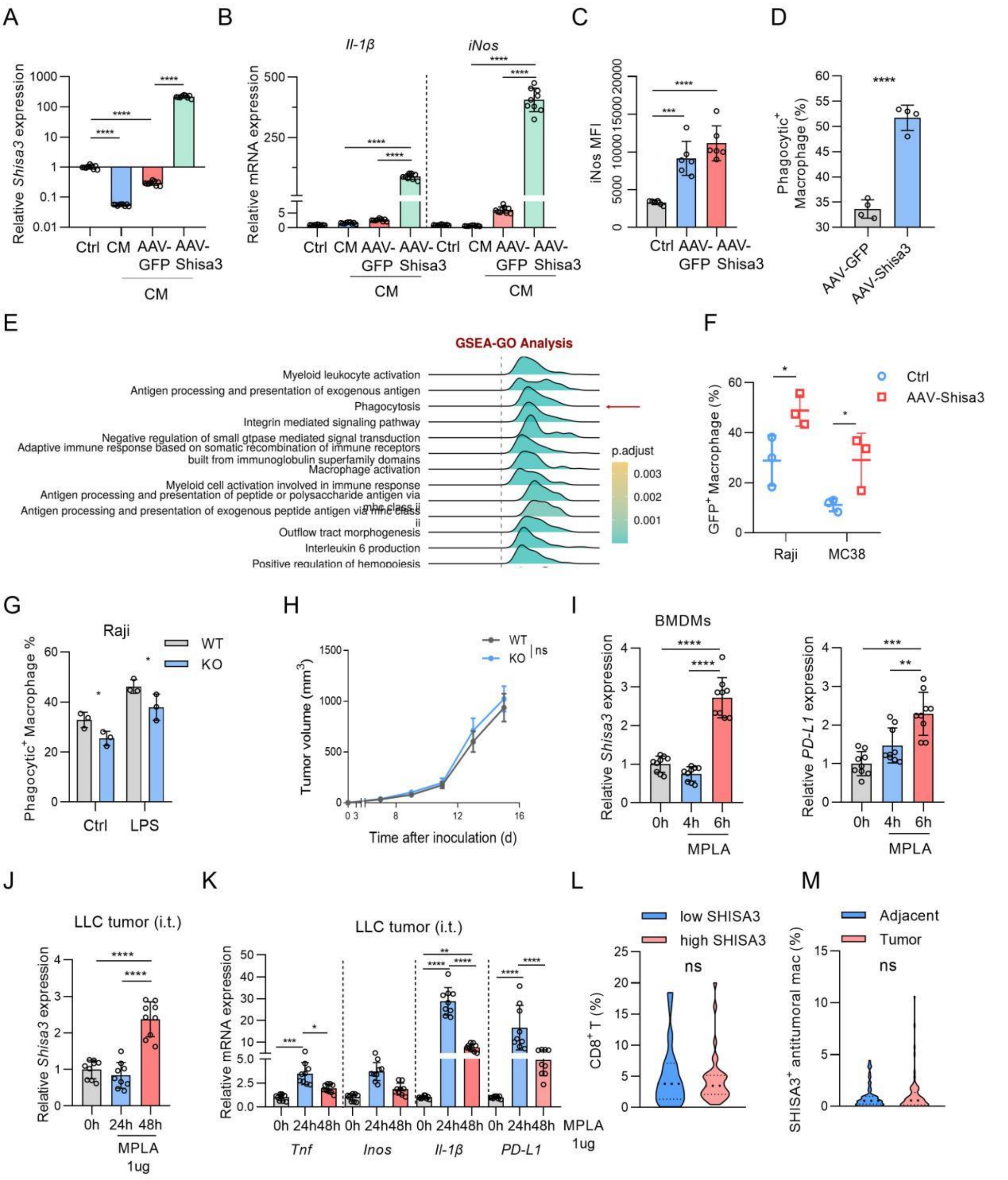


**Figure S8. SHISA3 promotes the phagocytic function of macrophages. A)** qRT-PCR of *Shisa3* expression in control, AAV-Shisa3 or AAV-GFP transduced BMDMs treated with or without 4T1 cell-CM. **B**) qRT-PCR of M1 markers *Il-1β* and *iNos* in control BMDMs, AAV-Shisa3 or AAV-GFP transduced BMDMs after treatment with 4T1 cell-CM. **C**) Scatterplots represent iNos expression in control BMDMs, AAV-Shisa3 or AAV-GFP transduced BMDMs after treatment with LLC cell-CM. **D**) Scatterplots represent percentages of the phagocytic function of control, AAV-Shisa3 or AAV-GFP transduced BMDMs for latex beads. **E)** GSEA-Gene Ontology (GO) analysis of SHISA3 related biological processes by BEST website (https://rookieutopia.com/app_direct/BEST/). **F)** Representative Scatterplots represent percentages of phagocytosis of control, AAV-Shisa3 or AAV-GFP transduced BMDMs for CFSE labeled tumor cells (Raji or MC-38). **G**) Scatterplots represent percentages of phagocytosis of WT BMDMs and Shisa3-KO BMDMs for CellTrace™ Far Red labeled tumor cells (Raji). **H)** LLC tumor growth on day 15 after tumor inoculation in WT and

Shisa3-KO mice. **I)** qRT-PCR of *Shisa3* or *PD-L1* mRNA expression in BMDMs after being treated with MPLA for different time. **J, K)** qRT-PCR of Shisa3 (**J**) or M1 markers expression (**K**) in LLC tumors treated with MPLA by intratumoral injections for 24h, 48h. **L)** The frequencies of $CD8^{+}$ T cells comparing high to low levels of SHISA3 in 71 patients with NSCLC. **M)** The frequencies of $CD68^{+}$ $CD163^{-}$ $SHISA3^{+}$ antitumoral macrophages comparing lung cancer and adjacent tissues. Data are presented as mean ± SD and were analyzed by one-way ANOVA (**A-C and I-K)** or unpaired two-tailed t-test (**D, F, L, M**) or two-way ANOVA with a mixed-effects model and adjusted by Holm–Šídák post-hoc test (**H**). *p < 0.05, **p < 0.01, ***p < 0.001, ****p < 0.0001. Data are representative of at least three independent experiments.

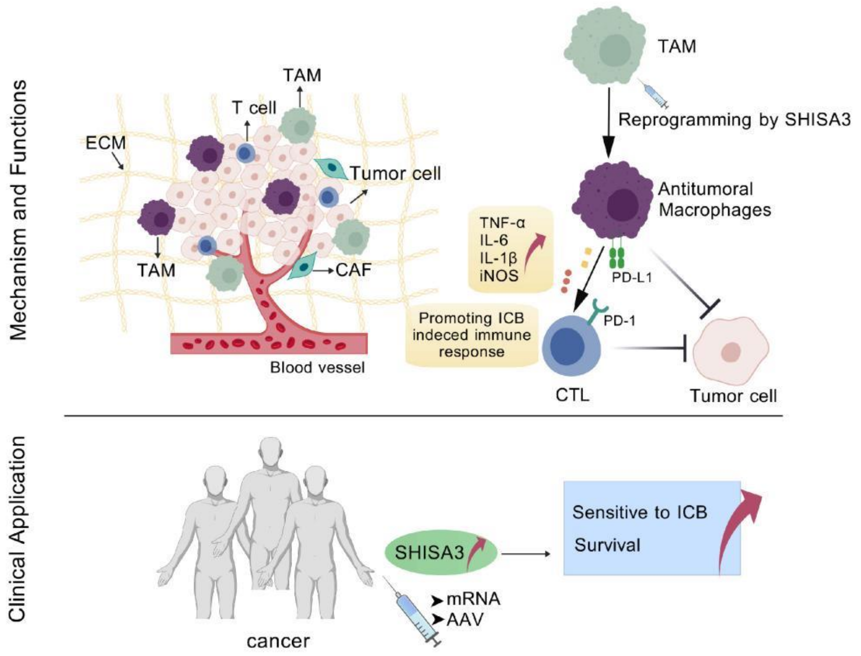


**Figure S9. A working model for how overexpression of SHISA3 reprograms TAMs and elicits potent anti-tumor immunity.**

**Supplementary Table S1. Primer sequences for Real-time PCR**

| **Primer** | **Sequence (5'->3')** |
|---|---|
| **Mouse genes** | |
| *Shisa2* | Forward: GCTACTGCTGCTCCAGCG |
| | Reverse: CACGTAGATGGGGACTGCC |
| *Shisa3* | Forward: TGTGCGGAGGTTCTCCTTTG |
| | Reverse: TGATACCAGGAAACCGGACG |
| *Shisa4* | Forward: TCGCCTCTGCTGTAATCCTC |
| | Reverse: TAGGAATCTCCTGGCCTTCAAAC |
| *Shisa5* | Forward: TCTGTTCGTCCGTCCGTTGG |
| | Reverse: CTTCACCAAAGGGCCTGCAC |
| *Shisa6* | Forward: AGCAGACTCCAGGTGATCGT |
| | Reverse:GAGCGTGAGAAGGAGAGGTC |
| *Shisa7* | Forward: CTCAGGCATCAAGCAGGACC |
| | Reverse:ATAGCACGCCAGTCGAGGTT |
| *Shisa8* | Forward: GCCTCAATAATGCGCCTCTG |
| | Reverse: GGTGCAACCTCTGTAGCCTT |
| *Shisa9* | Forward: TACCCCAACCTGGGTCAGAT |
| | Reverse: TTGACCTTGTCAGCTACTGC |
| *ShisaL1* | Forward: TGCAGGCAGTTGCTGGTG |
| | Reverse: AATGTGCGGACAGGACTGC |
| *ShisaL2a* | Forward: TACATGTGGTGGCTCAGCATCG |
| | Reverse: AGCTTGGTCTGAGGCTTTGAG |
| *ShisaL2b* | Forward: GCTTCGTGGAGCCTTTCCAG |
| | Reverse: CAACCAGAGCACCAATACTGAGG |
| *iNos* | Forward: CTATGGCCGCTTTGATGTGC |
| | Reverse: TTGGGATGCTCCATGGTCAC |
| *Mrc1* | Forward: TCATTCCCTCAGCAAGCGAT |
| | Reverse: GTGGATACTTGCCAGGTCCC |
| *Arg1* | Forward: GTAGACCCTGGGGAACACTAT |
| | Reverse: ATCACCTTGCCAATCCCCAG |

| | | |
|---|---|---|
| *Socs1* | Forward: TGGTTGTAGCAGCTTGTGTCTGG |
| | Reverse: CCTGGTTTGTGCAAAGATACTGGG |
| *Socs3* | Forward: GGGAGCCCCTTTGTAGACTT |
| | Reverse: CATCCCGGGGAGCTAGT |
| *Tnf* | Forward: AAGCCTGTAGCCCACGTCGT |
| | Reverse: GGCACCACTAGTTGGTTGTCTT |
| *Il-6* | Forward: TAGTCCTTCCTACCCCAATTTCC |
| | Reverse: TTGGTCCTTAGCCACTCCTTC |
| *Actin* | Forward: AGTGTGACGTTGACATCCGT |
| | Reverse: GCAGCTCAGTAACAGTCCGC |
| *Hspa8* | Forward: AGACCGTTACCAACGCTGTG |
| | Reverse: CCTTTCAGCTCCGACCTTCTT |
| **Human genes** | |
| SHISA3 | Forward: ACGCTGGACGCTACCATCTGCT |
| | Reverse: CCCAGGATGATGAACGCAATGAAG |
| GAPDH | Forward: TGATGACATCAAGAAGGTGG |
| | Reverse: TTGTCATACCAGGAAATGAGC |
| **ChIP-qPCR** | |
| CHIP-1 | Forward: AGTGTCCCAGGGAAAAGAACC |
| | Reverse: ATCAGTCCCCCAAGGGTCA |
| CHIP-2 | Forward: GAAGCGGGACCGTAGTCTT |
| | Reverse: CCTGCCACCTAGCTGTCACATA |
| **Shisa3-KO mice genotyping** | |
| PCR① | ATTATCAGCCTGCTGCCATCTCA |
| | TATCCATCTGGTTTGGCCTGATC |
| PCR② | TTTCCACTTGAGTTGGAACATCAG |
| | GTAAGTGGGGAGAATGCTCCAATC |
| **AAV Titration** | |
| ITR | GGAACCCCTAGTGATGGAGTT |
| | CGGCCTCAGTGAGCGA |
